# Effects of COVID-19-targeted non-pharmaceutical interventions on pediatric emergency department use: a quasi-experimental study interrupted time-series analysis in North Italian hospitals, 2017 to 2022

**DOI:** 10.3389/fpubh.2024.1439078

**Published:** 2024-07-31

**Authors:** Matteo Puntoni, Giuseppe Maglietta, Caterina Caminiti, Angela Miniaci, Marcello Lanari, Fabio Caramelli, Federico Marchetti, Alessandro De Fanti, Lorenzo Iughetti, Giacomo Biasucci, Agnese Suppiej, Andrea Miceli, Chiara Ghizzi, Gianluca Vergine, Melodie Aricò, Marcello Stella, Susanna Esposito, the Emilia-Romagna Paediatric COVID-19 network, Francesca Diodati, Chiara Maria Palo, Angela Miniaci, Luca Bertelli, Giovanni Biserni, Angela Troisi, Alessandra Iacono, Federico Bonvicini, Domenico Bartolomeo, Andrea Trombetta, Tommaso Zini, Nicoletta de Paulis, Cristina Forest, Battista Guidi, Francesca Di Florio, Enrico Valletta, Francesco Accomando, Greta Ramundo, Alberto Argentiero, Valentina Fainardi, Michela Deolmi

**Affiliations:** ^1^Clinical and Epidemiological Research Unit, University-Hospital of Parma, Parma, Italy; ^2^Pediatric Clinic, IRCCS Azienda Ospedaliera Universitaria di Bologna, Bologna, Italy; ^3^Pediatric Emergency Unit, IRCCS Azienda Ospedaliera Universitaria di Bologna, Bologna, Italy; ^4^Pediatric Intensive Care Unit, IRCCS Azienda Ospedaliera Universitaria di Bologna, Bologna, Italy; ^5^Pediatrics and Neonatology Unit, Ravenna Hospital, AUSL Romagna, Ravenna, Italy; ^6^Paediatrics Unit, Santa Maria Nuova Hospital, AUSL-IRCCS of Reggio Emilia, Reggio Emilia, Italy; ^7^Pediatrics Unit, Department of Medical and Surgical Sciences of Mothers, Children and Adults, University of Modena and Reggio Emilia, Modena, Italy; ^8^Pediatrics and Neonatology Unit, Guglielmo da Saliceto Hospital, Piacenza, Italy; ^9^Department of Medicine and Surgery, University of Parma, Parma, Italy; ^10^Pediatric Clinic, University of Ferrara, Ferrara, Italy; ^11^Pediatric Unit, Pavullo Hospital, AUSL Modena, Modena, Italy; ^12^Paediatrics Unit, Maggiore Hospital, Bologna, Italy; ^13^Pediatric Clinic, Rimini Hospital, AUSL Romagna, Rimini, Italy; ^14^Pediatric Unit, G.B. Morgagni – L. Pierantoni Hospital, AUSL Romagna, Forlì, Italy; ^15^Pediatric Unit, AUSL Romagna, Cesena, Italy; ^16^Pediatric Clinic, University-Hospital of Parma, Parma, Italy

**Keywords:** COVID-19 epidemiology, Non-Pharmaceutical Intervention, quasi-experimental design, Interrupted Time Series regression analysis, Diseases of the Respiratory System, mental disorders, injury and poisoning, Symptoms

## Abstract

**Background:**

The use of Non-Pharmaceutical Interventions (NPIs) during the COVID-19 pandemic is debated. Understanding the consequences these measures may have on vulnerable populations including children and adolescents is important.

**Methods:**

This is a multicenter, quasi-experimental before-after study involving 12 hospitals of the North Italian Emilia-Romagna Region, with NPI implementation as the intervention event. The 3 years preceding NPI implementation (in March 2020) constituted the pre-pandemic phase. The subsequent 2 years were further subdivided into a school closure phase (SC) and a subsequent mitigation measures phase (MM) with milder restrictions. Interrupted Time Series (ITS) regression analysis was used to calculate PED Standardized Incidence Rate Ratios (SIRR) on the diagnostic categories exhibiting the greatest frequency and/or variation.

**Results:**

In the 60 months of the study there were 765,215 PED visits. Compared to the pre-pandemic rate, overall PED presentations dropped by 58 and 39% during SC and MM, respectively. “Symptoms, signs and Ill-defined conditions,” “Injury and poisoning” and “Diseases of the Respiratory System” accounted for 74% of the reduction. A different pattern was instead seen for “Mental Disorders,” which exhibited the smallest decrease during SC, and is the only category which rose already at the end of SC. ITS analysis confirmed the strong decrease during SC (level change, IRR 0.17, 95%CI 0.12–0.27) and a significant increase in MM (slope change, IRR 1.23, 95%CI 1.13–1.33), with the sharpest decline (−94%) and rise (+36%) observed in the “Diseases of the Respiratory System” category. Mental Disorders showed a significant increasing trend of 1% monthly over the whole study period exceeding pre-pandemic levels at the end of MM. Females and adolescents showed higher increasing rates both in SC and MM.

**Conclusion:**

NPIs appear to have influenced PED attendance in different ways according to diagnostic categories, mirroring different mechanisms of action. These effects are beneficial in some cases and harmful in others, and establishing a clear balance between pros and cons is a difficult task for public health decision makers. The role of NPIs on PED use appropriateness deserves investigation. The rise in pediatric mental disorders independent of the pandemic makes interventions addressing these issues urgent.

## 1 Introduction

The COVID-19 pandemic has considerably affected healthcare systems, especially pediatric emergency care utilization (1, 2). In particular, an overall reduction of presentations of patients without COVID-19 and changes in disease patterns at the emergency service have been noted in recent research (3, 4). This phenomenon has been attributed to the uncertainty surrounding the infectivity and mortality rates of COVID-19, and to the consequent enforcement of strict lockdowns and other restrictive measures to reduce transmission (5), frequently defined as Non-Pharmaceutical Interventions (NPIs). To date, the debate on the need to apply population-wide NPIs is still open, also considering that COVID-19 in children and adolescents is usually mild or asymptomatic (6). For example, social distancing and school closure may have reduced the opportunities for the transmission of common infectious diseases, but they may have contributed to an increase in mental problems, as well as to the exacerbation of economic inequalities and domestic violence, and to educational setbacks (2, 7, 8). Therefore, it is essential to accurately measure the impact of NPIs on Pediatric Emergency Department (PED) use, to devise responses to future crises considering both benefits and harms. However, most studies on this topic are mainly descriptive, and only consider short time frames. Relatively few of these studies apply Interrupted Time Series (ITS) regression analysis, the recommended method to estimate the effectiveness of population-level health interventions that have been implemented at a clearly defined point in time (9). Our literature review conducted in Medline on March 27th, 2023 using strings for COVID-19, Interrupted Time Series, Emergency and Pediatric population, found 68 papers using ITS, of which 39 relevant to this topic. Each of the 39 studies (see Supplementary Appendix 1) exhibited at least one of four limitations which we considered important: (1) examined PED visits only for specific diagnoses (no. 29); (2) did not consider the age range of interest (no. 20); (3) was single-center (no. 15); and (4) considered a follow-up of <2 years (no. 36).

This study therefore aimed to quantify the impact of NPIs adopted during the COVID-19 pandemic on the trend of PED attendance in 12 hospitals in the Emilia-Romagna Region in Northern Italy, an area severely hit by COVID-19, during the 2 years following the start of the pandemic compared with the previous 3 years, considering two pandemic phases according to the type of adopted NPIs.

## 2 Materials and methods

### 2.1 Study design and setting

We conducted a multicenter, quasi-experimental controlled before-after study, aiming to estimate the change in PED attendance during the COVID-19 pandemic compared to the previous period. For disease categories exhibiting the greatest frequency and/or variations, we investigated the effect in different periods according to the intensity of NPIs. The setting and methodology of this study is the same described in detail in our previous paper (8), which focused on pediatric hospital admissions.

In 2020, the overall pediatric population in Emilia-Romagna amounted to 673,818 subjects (10), who were thus potentially affected by NPIs. The study spanned from March 2017 to February 2022 (60 months), defining the implementation of NPIs as an intervention event.

### 2.2 Intervention

The beginning of NPI implementation in Italy (national lockdown declared in March 2020) was used as delimitation. The previous 3 years were defined as the pre-COVID19 phase (PC), and the subsequent 2 years were further split into a school closure phase (SC, March to September 2020), and a mitigation measures phase (MM, October 2020 to February 2022), when schools were reopened but milder restrictions remained. School (including kindergarten) closure was chosen for its potentially more direct impact on young people compared to other NPIs.

### 2.3 Participants

Of the 15 PEDs of the Emilia-Romagna Region, 12 (80%) took part in the study, with an overall catchment area of 574,760 minor inhabitants in 2020 (corresponding to 85% of the total). Included subjects were patients aged between 0 and 17 years, presenting to the PED in the study period.

### 2.4 Data sources

Study data were anonymously gathered from routine electronic clinical records contained in the administrative databases of the Emilia-Romagna Regional Health Trust, and included the following: age, sex, admission dates, and diagnosis established after PED assessment –coded using the International Classification of Diseases, Ninth Revision, Clinical Modification (ICD-9-CM).

### 2.5 Statistical analysis

We examined monthly frequency of PED attendances, total and for ICD9-CM categories (the first three characters), during the 60 months considered by the study. To identify which major ICD9-CM categories had the greatest frequency and/or variation, the Standardized Incidence Rates (SIR) per 100,000 person-month were used. The adjusted or “standardized” rate is obtained by dividing the total of expected cases by the standard population. In this paper, “incidence measures” refer to the occurrence of PED attendance, since it was not possible to distinguish new cases from multiple accesses for the same child. To standardize estimates we considered the resident population in Europe in 2020 (the intermediate of the 5 years considered in this study) (11) and adjusting for age and sex. For each diagnostic category, we measured how any of the time periods changed with respect to the previous phase (SC vs. PC, MM vs. PC and MM vs. SC), by estimating the Standardized Incidence Rate Ratios (SIRR) and their 95% Confidence Intervals (95% CI). The SIRR is the estimate of the number of PED attendances in our population compared to the expected number based on the incidence rates in the European population. The SIRR was judged to be statistically significant if its 95% CI did not include 1. To investigate the effect of NPIs, the ICD9-CM categories exhibiting the greatest frequency/change were assessed using ITS regression analysis. Methods applied for ITS analysis were detailed previously (8). This segmented approach allows to estimate changes attributable to an intervention, in terms of overall (as time trend), immediate (as changes in level) and sustained (increase or decrease in the slope) effects, while accounting for pre-intervention secular trends. Post-hoc sensitivity analyses were conducted to investigate the impact of children aged 0–1 years old on IRR estimates from ITS modeling, since we assumed that a very small proportion of children in this age group attends day-care, an important factor since school closure is one of the main NPIs under study. All statistical analyses were centralized and performed with STATA (StataCorp. 2023. Stata Statistical Software: Release 18. College Station, TX: StataCorp LLC.).

## 3 Results

In the 60 months of the study, 765,215 PED visits were recorded. Table 1 compares case demographics for each of the three phases: PC, SC, and MM. In all phases, males were the majority, overall 56.2%, with the predominant age group being between 2 and 5 years (29.4%). We observed an average decrease of the Standardized Incidence Rate during school closure compared to the 3 years before the pandemic (1,361 vs. 3,218 × 100,000 person-month). Supplementary Table S1 shows the SIR and 95%CI by diagnostic category, highlighting that the reduction is generalized to all diagnoses.

**Table 1:** Demographics and PED attendance of the analyzed sample across the three phases.

	PC (Mar 1, 2017–Feb 28, 2020) *n* = 562,046		SC (Mar 1, 2020–Sep 30, 2020) *n* = 45,818		MM (Oct 1, 2020–Feb 28, 2022) *n* = 157,351		Whole period (Mar 1, 2017–Feb 28, 2022) *n* = 765,215	
Sex, *n (%) males*	314,421	(55.9)	26,232	(57.3)	88,998	(56.6)	429,651	(56.2)
Age class, *y n (%)*								
0–1	134,892	(24.0)	10,213	(22.3)	37,905	(24.1)	183,010	(23.9)
2–5	169,955	(30.2)	11,526	(25.2)	43,686	(27.8)	225,167	(29.4)
6–11	150,689	(26.8)	13,153	(28.7)	39,630	(25.2)	203,472	(26.6)
12–17	106,510	(19.0)	10,926	(23.9)	36,130	(23.0)	153,566	(20.1)
Standardized incidence rate, (95%CI)*	3,218 (3,168–3,267)		1,361 (1,329–1,394)		1,972 (1,932–2,012)		2,695.3 (2,649.4–2,741.3)	

*Rates are standardized × 100,000 (pop EU 2020). PED, Pediatric Emergency Department; PC, pre-COVID19 phase; SC, School closure phase; MM, Mitigation measures phase.

**Figure 1:**
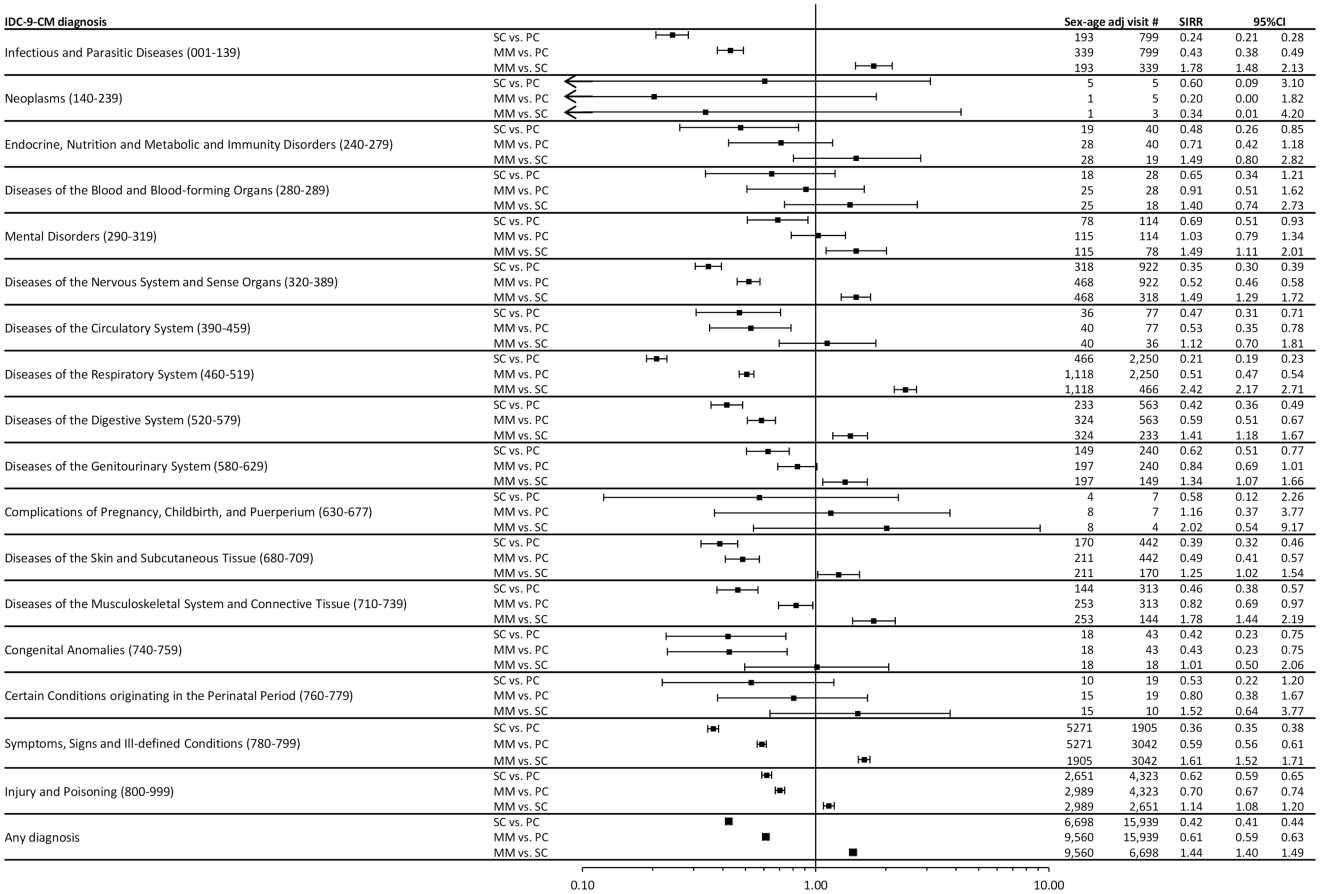
Forest plot of Standardized Incidence Rate Ratios (SIRR) for individual ICD9-CM diagnostic categories and for “any diagnosis,” during the three time phases: the 3 years pre-COVID19 phase (PC), the subsequent 1 year “school closure” phase (SC, March to September 2020), and the 1 year “mitigation measures” phase (MM, October 2020 to February 2022), when schools were reopened but milder restrictions remained. Estimates are reported as x 100,000 person-month and are age & sex standardized using as Standard the European resident population in 2020. SIRR, Standardized Incidence Rate Ratio; PC, pre-COVID19 phase; SC, School closure phase; MM, Mitigation measures phase.

Figure 1 displays the comparisons in terms of SIRR, overall and for individual ICD9-CM categories, between the three phases. Compared to the pre-pandemic rate, when schools were closed, overall PED presentations dropped by 58% (SIRR 0.42, 95%CI 0.41–0.44). It is evident that the greatest reduction was attributable to the following 3 diagnostic categories, which overall accounted for a share equal to 74% (6822/9241) of the reduction: “Symptoms, signs and Ill-defined conditions” (−33%, SIRR 0.67, 95%CI 0.64–0.70), “Injury and poisoning” (−38%, SIRR 0.62, 95%CI 0.59–0.65) and “Diseases of the Respiratory System” (−79%, SIRR 0.21, 95%CI 0.19–0.23).

Also during MM, with respect to PC, a less marked reduction was observed (−39%, SIRR 0.61, 95%CI 0.59–0.63), while a 44% overall increase (SIRR 1.44, 95%CI 1.40–1.49) was recorded with respect to SC, which however never restored data to pre-pandemic levels, a trend similar in most diagnoses. A different pattern was instead seen for “Mental Disorders,” which exhibited the smallest decrease during SC (SIRR 0.69, 95%CI 0.51–0.93), and is the only category featuring a substantial number of cases which rose back to pre-pandemic rates already at the end of SC (SIRR 1.03, 95%CI 0.79–1.34).

In the following sections, we present the model estimates from ITS regression analysis conducted on PED attendance for any diagnosis, and for the four above-mentioned categories, three of which were selected for their high frequency and one for its peculiar change patterns.

### 3.1 All diagnostic categories

**Figure 2:**
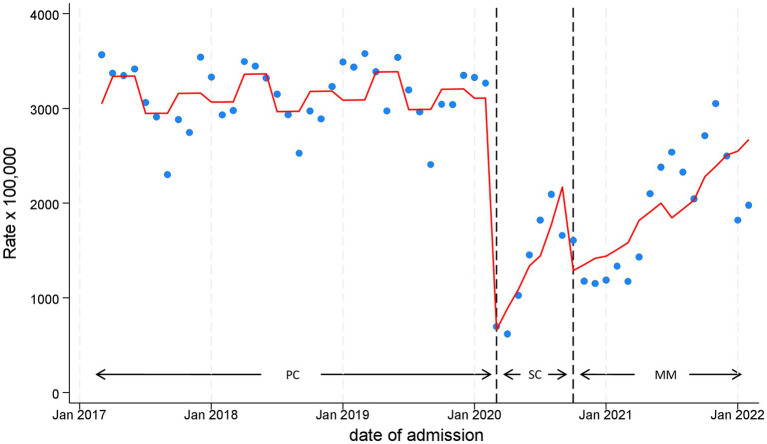
Monthly Pediatric Emergency Department Incidence Rate for any disease, with line trend from Interrupted Time Series (ITS) regression analysis during the three time phases: the 3 years pre-COVID19 phase (PC), the subsequent 1 year “school closure” phase (SC, March to September 2020), and the 1 year “mitigation measures” phase (MM, October 2020 to February 2022), when schools were reopened but milder restrictions remained. PC, pre-COVID19 phase; SC, School closure phase; MM, Mitigation measures phase.

Model estimates from ITS are shown in Table 2 and Figure 2. As evident, at the beginning of the school closure period there was a dramatic 82% decrease in visits per month (level change, IRR 0.17, 95%CI 0.12–0.27), compared to the pre-pandemic period. A similar decrease was also recorded in MM, although to a lesser extent (level change, IRR 0.38, 95%CI 0.31–0.47). Accordingly, a significant increase in trend was observed after the decrease particularly in SC (slope change, 23% per month, IRR 1.23, 95%CI 1.13–1.33), but also in MM (slope change, 5% per month, IRR 1.05, 95%CI 1.03–1.07). Rates seemed to tend toward pre-pandemic levels only in the autumn of 2021, 18 months after the pandemic started.

**Table 2:** Interrupted time series analysis results on PED attendance rates.

Variable	IRR	95%CI	*p*-value
*Level change^a^*			
SC vs. PC	0.17	0.12–0.27	<0.001
MM vs. PC	0.38	0.31–0.47	<0.001
MM vs. SC	2.19	1.40–3.43	0.001
*Slope change^b^*			
SC vs. PC	1.23	1.13–1.33	<0.001
MM vs. PC	1.05	1.03–1.07	<0.001
MM vs. SC	0.86	0.79–0.93	<0.001
*Time trend^c^*	1.00	0.99–1.01	0.792
*Season*			
Summer	1.00		
Winter	1.04	0.93–1.16	0.510
Spring	1.14	1.02–1.27	0.022
Autumn	1.07	0.96–1.19	0.218

^a^ Level change refers to an abrupt level change of the Incidence rate between the phases; ^b^ Slope change refers to slope change of the incidence rate over time between the phases; ^c^ Time trend refers to the change of Incidence rate associated with a time unit increase. PED, Pediatric Emergency Department; IRR, incidence rate ratio; PC, pre-COVID19 phase; SC, School closure phase; MM, Mitigation measures phase; 95%CI, 95% confidence interval. Pseudo *R*^2^ = 0.85. MM vs. SC contrast was manually added for interpretative purposes without *p*-value adjustment for multiple comparison.

### 3.2 Symptoms, signs and Ill-defined conditions

**Figure 3:**
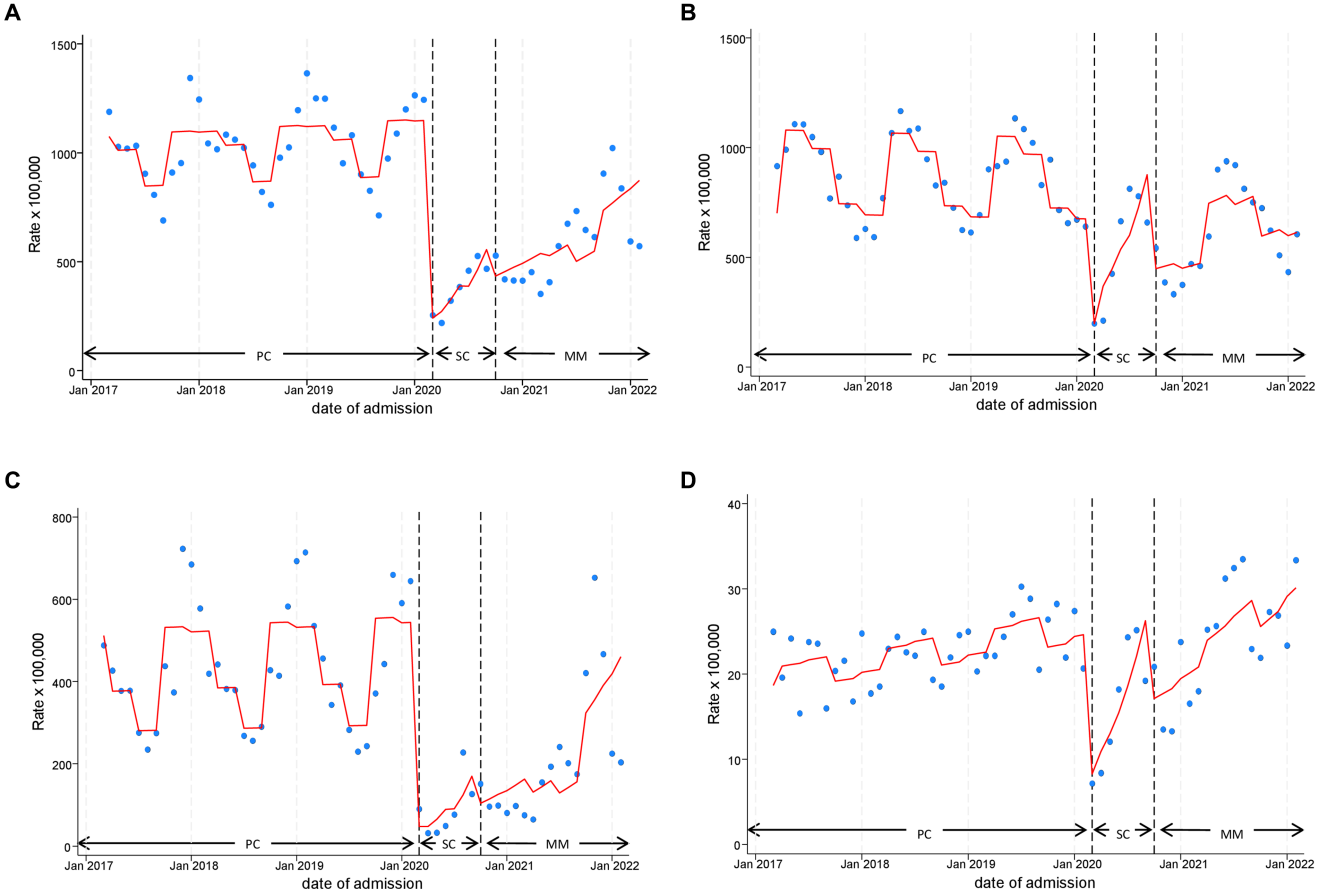
Monthly Pediatric Emergency Department Incidence Rate for Symptom, signs and Ill-defined conditions **(A)**, Injury and Poisoning **(B)**, Diseases of the Respiratory System **(C)** and Mental Disorders **(D)** with line trend from Interrupted Time Series (ITS) regression analysis during the three time phases: the 3 years pre-COVID19 phase (PC), the subsequent 1 year “school closure” phase (SC, March to September 2020), and the 1 year “mitigation measures” phase (MM, October 2020 to February 2022), when schools were reopened but milder restrictions remained. PC, pre-COVID19 phase; SC, School closure phase; MM, Mitigation measures phase.

Supplementary Table S2 features the most frequent subcategories of PED accesses included in this diagnostic category, consisting in nonspecific abnormal findings, and unknown causes of morbidity and mortality. The analysis displayed in Figure 3A highlights an overlapping time series with overall PED attendance shown in Figure 2. In particular, the beginning of SC was associated with an abrupt 82% drop of monthly visits (level change, IRR 0.18, 95%CI 0.11–0.28). After the first 2 months, visit counts followed a growing trend, recording an increase of 20% per month during this phase (slope change, IRR 1.20, 95%CI 1.09–1.31). A similar decrease was also observed in MM (level change, IRR 0.36, 95%CI 0.28–0.45), compared to the pre-pandemic period, with an increasing trend of 4% per month (slope change, IRR 1.04, 95%CI 1.02–1.06). We observed a statistically significant seasonality effect, with attendance rates at least 20% higher from autumn to spring compared to summer.

### 3.3 Injury and poisoning

The most frequent ICD-9CM diagnoses in this category are shown in Supplementary Table S3. A rapid decline during the onset of the pandemic was observed for this category (Figure 3B) as well, with a reduction in PED attendance of approximately 76% (level change, IRR 0.24, 95%CI 0.16–0.37). Similarly, we recorded a large increase in trend during SC (slope change, 21% per month, IRR 1.21, 95%CI 1.11–1.31). The reduction weakened in MM, showing −39% of PED presentations (level change, 0.61, 95%CI 0.48–0.78), with a corresponding monthly increase of 3% (slope change, IRR 1.03, 95%CI 1.01–1.05). In this category, we observed recurrent seasonality, also during the pandemic period, with spring and summer characterized by the highest attendance rates.

### 3.4 Diseases of the respiratory system

Supplementary Table S4 reports the most frequent ICD-9CM individual diagnoses in the category which mostly contributed to the abrupt decrease of overall PED attendance, both in SC (level change IRR 0.06, 95%CI 0.02–0.21) and in MM (IRR 0.17, 95%CI 0.10–0.28), compared to PC. The sharp decline (Figure 3C and Table 3) in SC corresponded to an equally sharp rise in MM slope change (IRR 1.36, 95%CI 1.09–1.72) which decreased in intensity in MM (IRR 1.10, 95%CI 1.06–1.14), compared to PC. The seasonality effect in this category was the strongest, with higher attendance rates especially in winter and autumn compared to summer.

**Table 3:** Interrupted time series analysis results on PED attendance rates for Symptoms, Signs and Ill-defined Conditions, Injury and Poisoning, Diseases of the Respiratory System and Mental Disorders.

	Symptoms, signs and Ill-defined conditions			Injury and poisoning			Diseases of the respiratory system			Mental disorders		
	IRR	95%CI	*p*-value	IRR	95%CI	*p*-value	IRR	95%CI	*p*-value	IRR	95%CI	*p*-value
*Level change^a^*												
SC vs. PC	0.18	0.11–0.28	<0.001	0.24	0.16–0.37	<0.001	0.06	0.02–0.21	<0.001	0.28	0.18–0.43	<0.001
MM vs. PC	0.36	0.28–0.45	<0.001	0.61	0.48–0.78	<0.001	0.17	0.10–0.28	<0.001	0.65	0.52–0.82	<0.001
MM vs. SC	2.02	1.24–3.32	0.005	2.52	1.59–4.00	<0.001	2.61	0.74–9.26	0.137	2.32	1.48–3.65	<0.001
*Slope change^b^*												
SC vs. PC	1.20	1.09–1.31	<0.001	1.21	1.11–1.31	<0.001	1.36	1.09–1.72	0.008	1.18	1.09–1.28	<0.001
MM vs. PC	1.04	1.02–1.06	<0.001	1.03	1.01–1.05	0.014	1.10	1.06–1.14	<0.001	1.03	1.01–1.04	0.004
MM vs. SC	0.87	0.79–0.96	0.005	0.85	0.78–0.92	<0.001	0.80	0.64–1.01	0.065	0.87	0.80–0.95	0.001
*Time trend^c^*	1.00	0.99–1.01	0.414	1.00	0.99–1.01	0.669	1.00	0.99–1.01	0.688	1.01	1.00–1.01	0.005
*Season*												
Summer	1.00			1.00			1.00			1.00		
Winter	1.28	1.13–1.44	<0.001	0.70	0.62–0.80	<0.001	1.84	1.44–2.33	<0.001	0.89	0.78–1.01	0.074
Spring	1.20	1.06–1.36	0.004	1.08	0.96–1.21	0.186	1.35	1.04–1.74	0.023	0.99	0.87–1.13	0.868
Autumn	1.29	1.14–1.45	<0.001	0.75	0.66–0.85	<0.001	1.88	1.48–2.39	<0.001	0.86	0.76–0.98	0.026

^a^ Level change refers to an abrupt level change of the Incidence rate between the phases; ^b^ Slope change refers to slope change of the incidence rate over time between the phases; ^c^ Time trend refers to the change of Incidence rate associated with a time unit increase. PED, Pediatric Emergency Department; IRR, incidence rate ratio; PC, Pre-COVID19 phase; SC, School closure phase; MM, Mitigation Measures phase; 95%CI, 95% confidence interval. Pseudo *R*^2^ = 0.84 for Signs and Ill-defined Conditions, 0.77 for Injury & Poisoning model, 0.78 for Diseases of the Respiratory System model; 0.30 for Mental Disorders. MM vs. SC contrasts were manually added for interpretative purposes without *p*-value adjustment for multiple comparison.

### 3.5 Mental disorders

Unlike the previously examined categories, Mental Disorders (Figure 3D and Table 3) exhibited a significant increasing trend over the whole study period, approximately 1% monthly (time trend IRR: 1.01, 95%CI 1.00–1.01). In fact, although a significant reduction was observed at the beginning of lockdown (SC level change, IRR 0.28, 95%CI 0.18–0.43), occurrence of attendance was restored to pre-COVID19 levels in the following months of SC and MM (slope change, IRR 1.18, 95%CI 1.09–1.28 and 1.03, 95%CI 1.01–1.04, respectively). This increase eventually led to levels exceeding pre-pandemic ones at the end of MM. The seasonality component analysis showed higher attendance rates during spring and summer. The list of most frequent diagnoses included in this category is provided in Supplementary Table S5.

### 3.6 Subgroup analysis by sex and age

Supplementary Tables S6–S9 and Supplementary Figure S1, S2 report the most relevant findings of subgroup analyses. Overall, the 0–5 age range exhibited the largest persistent reduction of PED attendance up to MM (level change, IRR 0.33, 95%CI 0.26–0.43, Supplementary Table S7). In the Mental Disorders category, subgroup analysis by sex (Supplementary Table S8) showed a sharper increasing trend (22% for month) for women in SC (slope change, IRR 1.22, 95%CI 1.11–1.33), and quite sustained in MM (slope change, IRR 1.04, 95%CI 1.02–1.06). Regarding age (Supplementary Table S9), the 12–17 class experienced the most abrupt decline at the beginning of lockdown (level change, IRR 0.26, 95%CI 0.16–0.43) and the most drastic increase during SC (slope change, IRR 1.21, 95%CI 1.10–1.33). This monthly increase persisted in a statistically significant manner also during the 17 months of MM (slope change, IRR 1.03, 95%CI 1.01–1.05) only in this age class. No statistically significant differences were observed in the overall analysis by sex (Supplementary Table S6), and in the Symptom, signs and Ill-defined conditions, Injury and Poisoning, and Diseases of the Respiratory System diagnostic categories (data not shown).

### 3.7 Sensitivity analyses

As shown in Supplementary Tables S10, S11, our analysis indicated that the exclusion of the 0 to 1 age class had a negligible impact on results.

## 4 Discussion

This study shows the effects on PED utilization of NPIs with different intensity, enforced during the COVID-19 pandemic in an Italian area severely impacted by COVID-19. To our knowledge, no multicenter study with this aim has been conducted considering an extended 2-year pandemic period, and applying rigorous ITS regression.

Overall, we recorded a drastic drop in PED presentations during the first 3 months of NPI implementation, when the number of monthly pre-pandemic visits was less than one third of that recorded before the pandemic (Figure 1). These findings are in line with those obtained in the systematic review by Roland et al. (5), who reported a mean percentage change in PED visits across 69 included studies, with a reduction of 63.86% (95% CI 60.40 to 67.31%) with respect to a corresponding pre-pandemic time period. After the initial brisk drop, we observed an overall increase, although this was slow and overall PED attendance rates were never restored to pre-pandemic levels in the two pandemic years we investigated. However, based on our ITS analysis, the direction of the upward trend in MM (1.05 slope change) is such to support the hypothesis that pre-pandemic values will eventually be reached. We do not know whether this trend was observed in other contexts, since we could not find published studies covering such a long follow-up period applying ITS analysis.

Our analysis revealed differences in the trends of PED use, after an initial collapse, between the individual diagnostic categories considered in this study, which enable us to formulate a number of hypotheses. For instance, the “Symptoms, signs and Ill-defined conditions” category, generally comprising low acuity, non-urgent conditions, exhibited the slowest increase compared to the other categories. This could reflect an improved awareness of the actual need for emergency care, and the tendency to manage less severe problems at home even after restrictions had been loosened, a phenomenon also emphasized in previous research (5, 12, 13). However, we cannot rule out the existence of unmet healthcare needs caused by real or perceived barriers to healthcare access. The interpretation of observed trends for “Diseases of the Respiratory System” leads to different considerations, more specific than those concerning the former category. In this case, PED attendance remained significantly reduced for a very extended period (18 months), and started to rise only in autumn 2021. This could be explained by a possible synergistic effect produced by NPIs (such as social distancing and the use of masks and other protective equipment), which produced significant changes in disease self-management and general behavior among chronic respiratory patients, leading to a reduction in the spread of common respiratory viruses, and reduced exposure to pollution and allergens (1, 2, 14). In this regard, the systematic review and meta-analysis by Kouis et al. (14), conducted only on observational cohort studies, found that asthma symptoms control (exacerbations, frequency and symptom severity) in children was significantly improved during lockdown. A further different pattern was observed for the “Injury and Poisoning” diagnostic category, with a more rapid return to pre-pandemic levels. This may be explained by the limited time children spent out-doors or practicing sports as a result of restrictions, which may have led to a decrease in emergency visits for traumatic injuries, the most common PED visits in this category (1, 13). The trend in PED visits for “Mental Disorders” merits particular consideration, as for this diagnostic category PED attendance was restored to pre-pandemic levels already in SC, and eventually exceeded them in the following months during MM. Our data highlighted that the increase particularly affected adolescent females, in line with previous research, including our work on hospital admissions (8, 15). The possible detrimental effects of social distancing, school closure, and isolation on children and adolescents have been widely reported, and may partially account for these findings (16). However, it must be pointed out that in our study, the rise in PED visits for mental health was present also before the pandemic, indicating that probably other factors in addition to COVID-19 are negatively impacting on young people’s mental health, especially on female teenagers. Our data are in line with the steady increase in the prevalence of mental disorders in young people recorded during the last 20 years (17). This is becoming a public health priority, considering that, according to the World Health Organization (WHO), 1 in 7 adolescents (10–19 years of age) experiences mental disorders (18), which may have negative consequences into adulthood if not properly addressed.

With this study, like with our previous research on hospital admissions (8), we attempted to inform the ongoing debate on the impact and usefulness of NPIs during pandemic outbreaks, although we are aware of the difficulty to discriminate the effects of individual NPIs, which were often enacted simultaneously. Moreover, the success of pandemic responses depends on a wide range of factors, which complicate pandemic management for public health. Human interaction and behavior can affect the spreading of infectious diseases, and infectious diseases in turn lead to changes in our actions. Understanding this “feedback loop” is a key challenge of epidemiology, and is necessary to develop effective strategies against pandemics (19). Research based on mathematical models and computer simulations highlights varying performances of different pandemic intervention policies according to contextual characteristics such as population density, population mobility, size of the area in question, and the type of pathogen (20). Taken together, these observations indicate that one-fits-all NPI strategies are not recommended, requiring policymakers to tailor their approach to individual populations and contexts (19). This process should also attempt to limit potential undesirable effects NPIs may cause. As for school closure, the literature emphasizes the uncertainty of its effectiveness in containing COVID-19 transmission in the community (21), as well as the numerous negative consequences on the pediatric population, such as the disruption of education, lack of various school-based services, an increase of mental health problems and increase in sedentary behavior (22, 23). A Cochrane systematic review, updated in 2023 to include COVID-19 research (24), investigated the effectiveness of various physical interventions on the spread of viruses. Lack of effectiveness was reported for mask wearing, a finding for which the authors postulated different explanations: problems with study design, low viral circulation, inappropriate use of masks (low adherence, extended use, self-contamination), lack of eye protection, and exaggerated sense of security. A modest effect was instead reported for hand hygiene, and its role as an essential component of other interventions was stressed (24). Hygienic measures appeared to be particularly effective in younger children. Acting on this population is important, as they can transmit infection in their household. However, the authors of the review also emphasized the high heterogeneity and poor reporting of studies on hand washing, which may limit confidence. The authors concluded that public health measures and physical interventions can be mostly effective in interrupting the spread of respiratory viral infections when they are delivered in combination as part of a structured program including education. Various knowledge gaps remain, both on the appropriate type and intensity of intervention combinations, and on the effectiveness of individual NPIs, which should be addressed, when possible in large, randomized trials with a pragmatic design.

We are aware that the existence of multiple COVID-19 strains may have influenced the effectiveness of NPIs and consequent public health policies, given their different transmission rates, severity levels, and vaccine responses (25). However, the investigation of the potential role of variants was not an objective of this work, since we did not analyze cases of COVID-19. Furthermore, in Italy, after the initial period of nationally imposed restrictions, the type and intensity of NPIs varied locally, determined by periodic epidemiological risk assessments by the Ministry of Health which assigned each region/province to one of three progressively restrictive tiers (26). These assessments considered 21 indicators on transmissibility, burden on the older adults, and healthcare, and resilience of monitoring systems. Therefore, a clearcut association between changes in the NPI strategy and the predominance of individual variants (27) is not easily determined.

This study has some strengths. The analysis concerned data provided by numerous centers that were critically affected by the pandemic, and are not restricted to specific diagnoses, therefore offering a complete picture of PED use before and during the pandemic. Furthermore, the use of ITS regression is a distinguishing characteristic of our work, since it enables to track a long-term period before and after a point of intervention to assess the intervention’s effects. Finally, the extended study period (24 months into the pandemic) enabled to investigate NPI effects in the long-term, allowing to make some considerations that may be useful for decision makers.

Some limitations should also be acknowledged. Firstly, we relied on routine data, which were not collected with epidemiologic purposes. Therefore, it was not possible to measure disease incidence or to make causal inferences on observed trend changes. Given the nature of our data, the risk of diagnostic misclassification should also be acknowledged. Secondly, the unavailability of severity codes in our data set prevented us to verify to which extent PED attendance reduction was due to a decrease in low-acuity health problems, possibly not requiring emergency medicine visits, or whether it was caused by an unmet healthcare need. Thirdly, we did not collect data on socioeconomic status, ethnicity, and gender identity, which would have helped us to understand whether the impact was stronger for more vulnerable populations.

## 5 Conclusion

NPIs enforced during the COVID-19 pandemic affected PED utilization in various ways, depending on individual diagnostic categories, mirroring different mechanisms of action. These effects appear to be beneficial in some cases and harmful in others, and establishing a clear balance between pros and cons is a difficult task for public health decision makers. It is well documented that NPIs have had a wide range of adverse effects which varied between different groups, depending on gender, age, socioeconomic status, lifestyle habits or health status, and country/region (28). The wide gaps in currently available evidence call for more targeted research aiming to better understand the consequences of NPIs on children and adolescents, also considering public acceptability and attitudes toward individual interventions. The possible change in behavior that our data seem to suggest concerning PED use for less severe conditions should be further investigated, to elucidate the extent to which it reflects a decrease of inappropriate emergency service attendance, and a failure to seek medical help for urgent health problems. This knowledge should form the basis for clear indications that should be provided to citizens, not only during health crises, in order to improve the use of emergency medicine, which constitutes a longstanding major public health problem (29). Finally, particular attention should be given to the early identification and proper management of mental health issues in young people, keeping in mind that this health emergency exists, and is worsening, regardless of the pandemic. The relevance of the problem, for the number of affected individuals and for the potential consequences that can persist into adulthood if untreated, stresses the need for immediate action in this regard.

## Data availability statement

The data analyzed in this study is subject to the following licenses/restrictions: the raw data supporting the conclusions of this article will be made available by the authors upon a motivated request to the corresponding author. Requests to access these datasets should be directed to ccaminiti@ao.pr.it.

## Ethics statement

The studies involving humans were approved by Area Vasta Emilia Nord (AVEN) Ethics Committee. The studies were conducted in accordance with the local legislation and institutional requirements. The ethics committee/institutional review board waived the requirement of written informed consent for participation from the participants or the participants’ legal guardians/next of kin because of feasibility issues (nearly 800,000 subjects should have been contacted). Waiver was provided by the Italian Data Protection Authority (Garante della Privacy).

## Author contributions

MP: Conceptualization, Data curation, Formal analysis, Investigation, Methodology, Software, Supervision, Validation, Visualization, Writing – original draft, Writing – review & editing. GM: Conceptualization, Formal analysis, Investigation, Methodology, Software, Supervision, Validation, Visualization, Writing – original draft, Writing – review & editing. CC: Conceptualization, Investigation, Methodology, Project administration, Software, Supervision, Validation, Visualization, Writing – original draft, Writing – review & editing. AMin: Investigation, Writing – original draft, Writing – review & editing, Resources. ML: Investigation, Writing – original draft, Writing – review & editing, Resources. FC: Investigation, Writing – original draft, Writing – review & editing, Resources. FM: Investigation, Writing – original draft, Writing – review & editing, Resources. AF: Investigation, Writing – original draft, Writing – review & editing, Resources. LI: Investigation, Writing – original draft, Writing – review & editing, Resources. GB: Investigation, Writing – original draft, Writing – review & editing, Resources. AS: Investigation, Writing – original draft, Writing – review & editing, Resources. AMic: Investigation, Writing – original draft, Writing – review & editing, Resources. CG: Investigation, Writing – original draft, Writing – review & editing, Resources. GV: Investigation, Writing – original draft, Writing – review & editing, Resources. MA: Investigation, Writing – original draft, Writing – review & editing, Resources. MS: Investigation, Writing – original draft, Writing – review & editing, Resources. SE: Investigation, Project administration, Supervision, Writing – original draft, Writing – review & editing, Conceptualization, Resources.

## Group members of the Emilia-Romagna Paediatric COVID-19 network

Collective authors who fulfill all four ICMJE authorship criteria.

Francesca Diodati, Chiara Maria Palo: Clinical and Epidemiological Research Unit, University Hospital of Parma, Parma, Italy; Angela Miniaci, Luca Bertelli: Pediatric Clinic, IRCCS Azienda Ospedaliera Universitaria di Bologna, Bologna, Italy; Giovanni Biserni: Pediatric Emergency Unit, IRCCS Azienda Ospedaliera Universitaria di Bologna, Bologna, Italy; Angela Troisi, Alessandra Iacono: Pediatrics and Neonatology Unit, Ravenna Hospital, AUSL Romagna, Ravenna, Italy; Federico Bonvicini, Domenico Bartolomeo, Andrea Trombetta: Paediatrics Unit, Santa Maria Nuova Hospital, AUSL-IRCCS of Reggio Emilia, Reggio Emilia, Italy; Tommaso Zini: Pediatrics Unit, Department of Medical and Surgical Sciences of Mothers, Children and Adults, University of Modena and Reggio Emilia, Modena, Italy; Nicoletta de Paulis: Pediatrics and Neonatology Unit, Guglielmo da Saliceto Hospital, Piacenza, Italy; Cristina Forest: Pediatric Clinic, University of Ferrara, Ferrara, Italy; Battista Guidi: Pediatric Unit, Pavullo Hospital, AUSL Modena, Pavullo, Italy; Francesca Di Florio: Paediatrics Unit, Maggiore Hospital, Bologna, Italy; Enrico Valletta, Francesco Accomando: Pediatric Unit, G.B. Morgagni – L. Pierantoni Hospital, AUSL Romagna, Forlì; Greta Ramundo, Alberto Argentiero, Valentina Fainardi, Michela Deolmi: Pediatric Clinic, University Hospital, Department of Medicine and Surgery, University of Parma, Parma, Italy.

## Supplementary material

SUPPLEMENTARY FIGURE S1 Monthly rate of PED attendance rate, with line trend from with line trend from Interrupted Time Series (ITS) regression analysis during the three time phases: the three years pre-COVID19 phase (PC), the subsequent one year “school closure” phase (SC, March to September 2020), and the one year “mitigation measures” phase (MM, October 2020 to February 2022), when schools were reopened but milder restrictions remained, by sex **(A)** and age category **(B)**. PED, Pediatric Emergency Department; PC, pre-COVID19 phase; SC, School closure phase; MM, Mitigation measures phase.

SUPPLEMENTARY FIGURE S2 Monthly rate of PED attendance rate for Mental Disorders with line trend from Interrupted Time Series (ITS) regression analysis during the three time phases: the three years pre-COVID19 phase (PC), the subsequent one year “school closure” phase (SC, March to September 2020), and the one year “mitigation measures” phase (MM, October 2020 to February 2022), when schools were reopened but milder restrictions remained, by sex **(A)** and age category **(B)**. PED, Pediatric Emergency Department; PC, pre-COVID19 phase; SC, School closure phase; MM, Mitigation measures phase.
