# Supplementary material for: Effects of COVID-19-targeted non-pharmaceutical interventions on pediatric emergency department use: a quasi-experimental study interrupted time-series analysis in North Italian hospitals, 2017 to 2022
Source: Front Public Health. 2024 Jul 31;12:1439078. doi: 10.3389/fpubh.2024.1439078 (PMC11322479; doi:10.3389/fpubh.2024.1439078)
Supplement: Supplementary file 1 [file Table_1.DOCX]

**Supplementary tables**

Supplementary Table S1: Monthly standardized incidence rates (SIR) (95%CI) x 100,000 (pop EU 2020) and total number, by type of diagnosis.

Supplementary Table S2: Type of Symptoms, signs and ill-defined conditions, sorted by frequency (>1% on total, N=246,893).

Supplementary Table S3: Type of Injury and poisoning diagnosis, sorted by frequency (>1% on total, N=223,551).

Supplementary Table S4: Type of Diseases of the Respiratory System, sorted by frequency (>1% on total, N=98,396).

Supplementary Table S5: Type of Mental disorders diagnosis, sorted by frequency (>1% on total, N=6,575).

Supplementary Table S6: Interrupted time series analysis results on PED attendance rates, by sex.

Supplementary Table S7: Interrupted time series analysis results on PED attendance rates, by age category.

Supplementary Table S8: Interrupted time series analysis results on PED attendance rates for Mental disorders, by sex.

Supplementary Table S9: Interrupted time series analysis results on PED attendance rates for Mental disorders, by age category.

Supplementary Table S10: Interrupted time series analysis results on PED attendance rates, excluding 0-1 years old subjects (sensitivity analysis).

Supplementary Table S11: Interrupted time series analysis results on PED attendance rates for Symptoms, signs and ill-defined conditions, Injury and poisoning, Diseases of the Respiratory System and Mental disorders, excluding 0-1years old subjects (sensitivity analysis).

| Supplementary Table S1. Monthly standardized incidence rates (SIR) (95%CI) x 100,000 (pop EU 2020) and total number, by type of diagnosis | | | | |
| --- | --- | --- | --- | --- |
|  | **SIR** | | | **Total number**  **(Mar 1, 2017-**  **Feb 28, 2022)** |
|  | **PC**  **(Mar 1, 2017-**  **Feb 28, 2020)** | **SC**  **(Mar 1, 2020-**  **Sep 30, 2020)** | **MM**  **(Oct 1, 2020-**  **Feb 28, 2022)** |  |
|  | *(n=562,046)* | *(n=45,818)* | *(n=157,351)* | *(N=765,215)* |
| Infectious and Parasitic Diseases (001-139) | 161 (150-173) | 39 (34-45) | 70 (62-77) | 34,561 (4.5%) |
| Neoplasms (140-239) | 1 (0-2) | 0 (0-1) | 0 (0-1) | 404 (0.1%) |
| Endocrine, Nutritional & Metabolic & Immunity Disorders (240-279) | 8 (6-11) | 4 (2-6) | 6 (4-8) | 1,994 (0.3%) |
| Diseases of the Blood and Blood-forming Organs (280-289) | 6 (4-8) | 4 (2-5) | 5 (3-7) | 1,638 (0.2%) |
| Mental Disorders (290-319) | 23 (19-27) | 16 (12-20) | 24 (19-28) | 6,575 (0.9%) |
| Diseases of the Nervous System and Sense Organs (320-389) | 186 (174-198) | 65 (58-72) | 96 (87-105) | 42,482 (5.6%) |
| Diseases of the Circulatory System (390-459) | 16 (12-19) | 7 (5-10) | 8 (6-11) | 3,663 (0.5%) |
| Diseases of the Respiratory System (460-519) | 454 (435-473) | 95 (86-104) | 230 (216-244) | 98,396 (12.9%) |
| Diseases of the Digestive System (520-579) | 114 (104-123) | 47 (41-54) | 67 (59-74) | 26,757 (3.5%) |
| Diseases of the Genitourinary System (580-629) | 49 (42-55) | 30 (25-35) | 40 (35-46) | 12,793 (1.7%) |
| Complications of Pregnancy, Childbirth, & Puerperium (630-677) | 1 (0-3) | 1 (0-2) | 2 (1-3) | 522 (0.1%) |
| Diseases of the Skin and Subcutaneous Tissue (680-709) | 89 (81-98) | 35 (29-40) | 43 (37-49) | 20,330 (2.7%) |
| Dis. of the Musculoskeletal System & Connective Tissue (710-739) | 63 (56-70) | 30 (25-35) | 45 (39-51) | 16,024 (2.1%) |
| Congenital Anomalies (740-759) | 9 (6-11) | 4 (2-5) | 4 (2-6) | 1,887 (0.2%) |
| Certain Conditions originating in the Perinatal Period (760-779) | 4 (2-6) | 2 (1-3) | 3 (1-5) | 985 (0.1%) |
| Symptoms, Signs and Ill-defined Conditions (780-799) | 1064 (1035-1093) | 387 (369-405) | 625 (602-648) | 246,893 (32.3%) |
| Injury and Poisoning (800-999) | 873 (847-899) | 539 (518-559) | 614 (592-636) | 223,551 (29.2%) |
| Abbreviations: SIR= standardized incidence rates; PC= Pre-COVID19 phase; SC= School closure phase; MM=Mitigation Measures phase; 95%CI: 95% confidence interval | | | | |

Supplementary Table S2: Type of Symptoms, Signs and Ill-defined conditions, sorted by frequency (>1% on total, N= 246,893).

| **Description (ICD-9-CM code)** | **N.** | **%** |
| --- | --- | --- |
| Other general symptoms (780.9) | 85,504 | 34.6 |
| Fever and other physiologic disturbances of temperature regulation (780.6) | 50,620 | 20.5 |
| Abdominal pain, unspecified site (789.00) | 21,740 | 8.8 |
| Vomiting alone (787.03) | 14,021 | 5.7 |
| Cough (786.2) | 7,834 | 3.2 |
| Dyspnea and respiratory abnormalities (786.0) | 4,763 | 1.9 |
| Syncope and collapse (780.2) | 4,343 | 1.8 |
| Headache (784.0) | 4,247 | 1.7 |
| Other symptoms involving skin and integumentary tissues (782.9) | 3,763 | 1.5 |
| General symptoms (780) | 3,208 | 1.3 |
| Abdominal pain, generalized (789.07) | 2,777 | 1.1 |
| Abdominal pain, periumbilic (789.05) | 2,633 | 1.1 |
| Chest pain, unspecified (786.50) | 2,395 | 1.0 |

Supplementary Table S3: Type of injury and poisoning diagnosis, sorted by frequency (>1% on total, N= 223,551).

| **Description (ICD-9-CM code)** | **N.** | **%** |
| --- | --- | --- |
| Other and unspecified injury of head, face and neck (959) | 21,470 | 9.6 |
| Concussion with no loss of consciousness (850.0) | 9,387 | 4.2 |
| Head injury, unspecified (959.01) | 6,970 | 3.1 |
| Contusion of face, scalp, and neck except eye(s) (920) | 6,146 | 2.8 |
| Contusion of finger (923.3) | 4,869 | 2.2 |
| Sprain of ankle, unspecified site (845.00) | 4,442 | 2.0 |
| Contusion of upper limb (923) | 4,123 | 1.8 |
| Intracranial injury of other and unspecified nature without mention of open intracranial wound, with no loss of consciousness (854.01) | 3,538 | 1.6 |
| Contusion of unspecified part of upper limb (923.9) | 3,152 | 1.4 |
| Closed fracture of middle or proximal phalanx or phalanges of hand (816.01) | 3,013 | 1.4 |
| Insect bite, nonvenomous of face, neck, and scalp except eye, without mention of infection (910.4) | 2,954 | 1.3 |
| Sprains and strains of ankle and foot (845) | 2,906 | 1.3 |
| Contusion of lower limb and of other and unspecified sites (924) | 2,809 | 1.3 |
| Open wound of face, unspecified site, without mention of complication (873.40) | 2,745 | 1.2 |
| Contusion of unspecified site (924.9) | 2,637 | 1.2 |
| Closed fracture of phalanx or phalanges of hand, unspecified (816.00) | 2,563 | 1.2 |
| Other sprains and strains of ankle (845.09) | 2,521 | 1.1 |
| Fracture of one or more phalanges of hand (816) | 2,489 | 1.1 |
| Closed fracture of one or more phalanges of foot (826.0) | 2,460 | 1.1 |
| Contusion of wrist (923.21) | 2,291 | 1.0 |

Supplementary Table S4: Type of Diseases of the Respiratory System, sorted by frequency (>1% on total, N= 98,396).

| **Description (ICD-9-CM code)** | **N.** | **%** |
| --- | --- | --- |
| Acute upper respiratory infections of unspecified site (465.9) | 20,499 | 20.8 |
| Acute pharyngitis (462) | 12,762 | 13.0 |
| Acute upper respiratory infections of other multiple sites (465.8) | 9,774 | 9.9 |
| Acute bronchitis (466.0) | 8,549 | 8.7 |
| Acute tonsillitis (463) | 6,308 | 6.4 |
| Acute nasopharyngitis (common cold) (460) | 6,281 | 6.4 |
| Influenza with other respiratory manifestations (487.1) | 3,856 | 3.9 |
| Acute bronciolitis due to other infectious organisms (466.19) | 3,493 | 3.6 |
| Acute laryngitis and tracheitis (464) | 2,967 | 3.0 |
| Asthma, unspecified (493.90) | 2,965 | 3.0 |
| Acute bronchospasm (519.11) | 2,366 | 2.4 |
| Bronchopneumonia organism unspecified (485) | 1,947 | 2.0 |
| Pneumonia organism unspecified (486) | 1,898 | 1.9 |
| Other and unspecified diseases of upper respiratory tract (478.9) | 1,505 | 1.5 |
| Bronchitis, not specified as acute or chronic (490) | 1,301 | 1.3 |

Supplementary Table S5: Type of mental disorders diagnosis, sorted by frequency (>1% on total, N= 6,575).

| **Description (ICD-9-CM code)** | **N.** | **%** |
| --- | --- | --- |
| Anxiety state unspecified (300.00) | 948 | 14.4 |
| Acute alcoholic intoxication in alcoholism, unspecified (303.00) | 610 | 9.3 |
| Panic disorder without agoraphobia (300.01) | 573 | 8.7 |
| Tension headache (307.81) | 511 | 7.8 |
| Eating disorder unspecified (307.50) | 489 | 7.4 |
| Other and unspecified special symptoms or syndromes not elsewhere classified (307.9) | 432 | 6.6 |
| Alcohol abuse, unspecified (305.00) | 304 | 4.6 |
| Excitative type psychosis (298.1) | 279 | 4.2 |
| Unspecified acute reaction to stress (308.9) | 140 | 2.1 |
| Anxiety disorder in conditions classified elsewhere (293.84) | 138 | 2.1 |
| Adjustment disorder with anxiety (309.24) | 132 | 2.0 |
| Unspecified nonpsychotic mental disorder (300.9) | 129 | 2.0 |
| Unspecified psychophysiological malfunction (306.9) | 110 | 1.7 |
| Other, mixed, or unspecified drug abuse, unspecified (305.90) | 85 | 1.3 |
| Anorexia nervosa (307.1) | 72 | 1.1 |

| Supplementary Table S6: Interrupted time series analysis results on PED attendance rates, by sex. | | | | | | |
| --- | --- | --- | --- | --- | --- | --- |
|  | **Males** | | | **Females** | | |
|  | ***IRR*** | ***95%CI*** | ***p-Value*** | ***IRR*** | ***95%CI*** | ***p-Value*** |
| *Level change^a^* |  |  |  |  |  |  |
| SC vs. PC | 0.17 | 0.11-0.26 | <0.001 | 0.18 | 0.12-0.27 | <0.001 |
| MM vs. PC | 0.39 | 0.31-0.49 | <0.001 | 0.37 | 0.30-0.46 | <0.001 |
| MM vs. SC | 2.26 | 1.44-3.54 | <0.001 | 2.10 | 1.34-3.30 | 0.001 |
|  |  |  |  |  |  |  |
| *Slope change^b^* |  |  |  |  |  |  |
| SC vs. PC | 1.23 | 1.13-1.34 | <0.001 | 1.22 | 1.12-1.32 | <0.001 |
| MM vs. PC | 1.05 | 1.03-1.07 | <0.001 | 1.05 | 1.03-1.07 | <0.001 |
| MM vs. SC | 0.85 | 0.78-0.92 | <0.001 | 0.86 | 0.79-0.94 | 0.001 |
|  |  |  |  |  |  |  |
| *Time trend^c^* | 1.00 | 0.99-1.01 | 0.747 | 1.00 | 0.99-1.01 | 0.842 |
|  |  |  |  |  |  |  |
| *Season* |  |  |  |  |  |  |
| Summer | 1.00 |  |  | 1.00 |  |  |
| Winter | 1.01 | 0.91-1.13 | 0.837 | 1.07 | 0.96-1.19 | 0.219 |
| Spring | 1.14 | 1.02-1.27 | 0.017 | 1.13 | 1.01-1.26 | 0.032 |
| Autumn | 1.05 | 0.94-1.17 | 0.351 | 1.09 | 0.98-1.22 | 0.110 |
|  |  |  |  |  |  |  |
| ^a^ Level change refers to an abrupt level change of the Incidence rate between the periods; ^b^ Slope change refers to slope change of the incidence rate over time between the periods. ^c^ Time trend refers to the change of Incidence rate associated with a time unit increase. Abbreviations: PED= Pediatric Emergency Department; PC= Pre-COVID19 phase; SC= School closure phase; MM= Mitigation Measures phase; IRR= Incidence rate ratio; 95%CI: 95% confidence interval. Pseudo R2=0.84 for Males model, 0.85 for Females model. Note: MM vs. SC contrasts were manually added for interpretative purposes without p-value adjustment for multiple comparison. | | | | | | |

| Supplementary Table S7: Interrupted time series analysis results on PED attendance rates, by age category. | | | | | | | | | |
| --- | --- | --- | --- | --- | --- | --- | --- | --- | --- |
|  | **0-5y** | | | **6-11y** | | | **12-17y** | | |
|  | ***IRR*** | ***95%CI*** | ***p-Value*** | ***IRR*** | ***95%CI*** | ***p-Value*** | ***IRR*** | ***95%CI*** | ***p-Value*** |
| *Level change^a^* |  |  |  |  |  |  |  |  |  |
| SC vs. PC | 0.17 | 0.10-0.29 | <0.001 | 0.17 | 0.12-0.25 | <0.001 | 0.18 | 0.12-0.28 | <0.001 |
| MM vs. PC | 0.33 | 0.26-0.43 | <0.001 | 0.42 | 0.34-0.51 | <0.001 | 0.49 | 0.39-0.61 | <0.001 |
| MM vs. SC | 1.91 | 1.10-3.31 | 0.021 | 2.40 | 1.59-3.63 | <0.001 | 2.70 | 1.72-4.24 | <0.001 |
|  |  |  |  |  |  |  |  |  |  |
| *Slope change^b^* |  |  |  |  |  |  |  |  |  |
| SC vs. PC | 1.21 | 1.10-1.34 | <0.001 | 1.22 | 1.13-1.32 | <0.001 | 1.25 | 1.15-1.35 | <0.001 |
| MM vs. PC | 1.06 | 1.04-1.08 | <0.001 | 1.03 | 1.01-1.05 | <0.001 | 1.03 | 1.02-1.05 | <0.001 |
| MM vs. SC | 0.88 | 0.79-0.97 | 0.011 | 0.84 | 0.78-0.91 | <0.001 | 0.83 | 0.77-0.90 | <0.001 |
|  |  |  |  |  |  |  |  |  |  |
| *Time trend^c^* | 1.00 | 0.99-1.01 | 0.833 | 1.00 | 0.99-1.01 | 0.327 | 1.00 | 0.99-1.01 | 0.547 |
|  |  |  |  |  |  |  |  |  |  |
| *Season* |  |  |  |  |  |  |  |  |  |
| Summer | 1.00 |  |  | 1.00 |  |  | 1.00 |  |  |
| Winter | 1.15 | 1.01-1.31 | 0.042 | 0.97 | 0.88-1.08 | 0.603 | 0.89 | 0.79-0.99 | 0.036 |
| Spring | 1.16 | 1.01-1.32 | 0.030 | 1.17 | 1.06-1.30 | 0.002 | 1.04 | 0.93-1.16 | 0.502 |
| Autumn | 1.24 | 1.09-1.40 | 0.001 | 0.87 | 0.79-0.97 | 0.011 | 0.93 | 0.83-1.04 | 0.229 |
|  |  |  |  |  |  |  |  |  |  |
| ^a^ Level change refers to an abrupt level change of the Incidence rate between the periods; ^b^ Slope change refers to slope change of the incidence rate over time between the periods. ^c^ Time trend refers to the change of Incidence rate associated with a time unit increase. Abbreviations: PED= Pediatric Emergency Department; PC= Pre-COVID19 phase; SC= School closure phase; MM= Mitigation Measures phase; IRR= Incidence rate ratio; 95%CI: 95% confidence interval. Pseudo R2=0.83 for 0-5y model, 0.85 for 6-11y model, 0.74 for 12-17y model. Note: MM vs. SC contrasts were manually added for interpretative purposes without p-value adjustment for multiple comparison. | | | | | | | | | |

| Supplementary Table S8: Interrupted time series analysis results on PED attendance rates for Mental disorders, by sex. | | | | | | |
| --- | --- | --- | --- | --- | --- | --- |
|  | **Males** | | | **Females** | | |
|  | ***IRR*** | ***95%CI*** | ***p-Value*** | ***IRR*** | ***95%CI*** | ***p-Value*** |
| *Level change^a^* |  |  |  |  |  |  |
| SC vs. PC | 0.33 | 0.19-0.56 | <0.001 | 0.25 | 0.16-0.40 | <0.001 |
| MM vs. PC | 0.68 | 0.50-0.92 | 0.013 | 0.64 | 0.50-0.82 | <0.001 |
| MM vs. SC | 2.09 | 1.18-3.71 | 0.012 | 2.55 | 1.57-4.15 | <0.001 |
|  |  |  |  |  |  |  |
| *Slope change^b^* |  |  |  |  |  |  |
| SC vs. PC | 1.13 | 1.02-1.26 | 0.020 | 1.22 | 1.11-1.33 | <0.001 |
| MM vs. PC | 1.01 | 0.98-1.03 | 0.554 | 1.04 | 1.02-1.06 | <0.001 |
| MM vs. SC | 0.89 | 0.80-0.99 | 0.032 | 0.85 | 0.78-0.93 | 0.001 |
|  |  |  |  |  |  |  |
| *Time trend^c^* | 1.01 | 1.00-1.02 | 0.007 | 1.01 | 1.00-1.01 | 0.031 |
|  |  |  |  |  |  |  |
| *Season* |  |  |  |  |  |  |
| Summer | 1.00 |  |  | 1.00 |  |  |
| Winter | 0.79 | 0.67-0.94 | 0.006 | 0.97 | 0.85-1.11 | 0.645 |
| Spring | 0.91 | 0.77-1.07 | 0.240 | 1.06 | 0.92-1.21 | 0.417 |
| Autumn | 0.80 | 0.67-0.94 | 0.008 | 0.92 | 0.80-1.05 | 0.211 |
|  |  |  |  |  |  |  |
| ^a^ Level change refers to an abrupt level change of the Incidence rate between the periods; ^b^ Slope change refers to slope change of the incidence rate over time between the periods. ^c^ Time trend refers to the change of Incidence rate associated with a time unit increase. Abbreviations: PED= Pediatric Emergency Department; PC= Pre-COVID19 phase; SC= School closure phase; MM=Mitigation Measures phase; IRR= Incidence rate ratio; 95%CI: 95% confidence interval. Pseudo R2=0.17 for Males model, 0.27 for Females model. Note: MM vs. SC contrasts were manually added for interpretative purposes without p-value adjustment for multiple comparison. | | | | | | |

| Supplementary Table S9: Interrupted time series analysis results on PED attendance rates for Mental disorders, by age category. | | | | | | | | | |
| --- | --- | --- | --- | --- | --- | --- | --- | --- | --- |
|  | **0-5y** | | | **6-11y** | | | **12-17y** | | |
|  | ***IRR*** | ***95%CI*** | ***p-Value*** | ***IRR*** | ***95%CI*** | ***p-Value*** | ***IRR*** | ***95%CI*** | ***p-Value*** |
| *Level change^a^* |  |  |  |  |  |  |  |  |  |
| SC vs. PC | 0.38 | 0.18-0.80 | 0.011 | 0.31 | 0.15-0.64 | 0.002 | 0.26 | 0.16-0.43 | <0.001 |
| MM vs. PC | 0.50 | 0.32-0.79 | 0.003 | 0.82 | 0.56-1.21 | 0.318 | 0.66 | 0.51-0.85 | 0.002 |
| MM vs. SC | 1.32 | 0.59-2.96 | 0.493 | 2.66 | 1.23-5.74 | 0.013 | 2.50 | 1.49-4.21 | 0.001 |
|  |  |  |  |  |  |  |  |  |  |
| *Slope change^b^* |  |  |  |  |  |  |  |  |  |
| SC vs. PC | 1.08 | 0.92-1.26 | 0.363 | 1.12 | 0.96-1.30 | 0.157 | 1.21 | 1.10-1.33 | <0.001 |
| MM vs. PC | 1.03 | 0.99-1.06 | 0.166 | 1.00 | 0.97-1.03 | 0.842 | 1.03 | 1.01-1.05 | 0.003 |
| MM vs. SC | 0.95 | 0.81-1.12 | 0.559 | 0.90 | 0.77-1.05 | 0.175 | 0.85 | 0.77-0.94 | 0.001 |
|  |  |  |  |  |  |  |  |  |  |
| *Time trend^c^* | 1.01 | 1.00-1.02 | 0.021 | 1.01 | 1.00-1.02 | 0.119 | 1.01 | 1.00-1.01 | 0.069 |
|  |  |  |  |  |  |  |  |  |  |
| *Season* |  |  |  |  |  |  |  |  |  |
| Summer | 1.00 |  |  | 1.00 |  |  | 1.00 |  |  |
| Winter | 0.96 | 0.74-1.23 | 0.721 | 1.02 | 0.81-1.28 | 0.871 | 0.85 | 0.73-0.98 | 0.024 |
| Spring | 1.18 | 0.92-1.50 | 0.187 | 1.24 | 0.99-1.55 | 0.057 | 0.91 | 0.78-1.05 | 0.196 |
| Autumn | 0.94 | 0.74-1.21 | 0.645 | 0.88 | 0.70-1.11 | 0.280 | 0.85 | 0.74-0.98 | 0.028 |
|  |  |  |  |  |  |  |  |  |  |
| ^a^ Level change refers to an abrupt level change of the Incidence rate between the periods; ^b^ Slope change refers to slope change of the incidence rate over time between the periods. ^c^ Time trend refers to the change of Incidence rate associated with a time unit increase. Abbreviations: PED= Pediatric Emergency Department; PC= Pre-COVID19 phase; SC= School closure phase; MM=Mitigation Measures phase; IRR= Incidence rate ratio; 95%CI: 95% confidence interval. Pseudo R2=0.08 for 0-5y model, 0.10 for 6-11 model, 0.28 for 12-17y model. Note: MM vs. SC contrasts were manually added for interpretative purposes without p-value adjustment for multiple comparison. | | | | | | | | | |

| Supplementary Table S10: Interrupted time series analysis results on PED attendance rates, excluding 0-1 years old subjects (sensitivity analysis). | | | |
| --- | --- | --- | --- |
| **Variable** | **HRR** | **95%CI** | **p-Value** |
|  |  |  |  |
| *Level change^a^* |  |  |  |
| SC vs. PC | 0.17 | 0.11-0.25 | <0.001 |
| MM vs. PC | 0.39 | 0.31-0.48 | <0.001 |
| MM vs. SC | 2.32 | 1.48-3.63 | <0.001 |
|  |  |  |  |
| *Slope change^b^* |  |  |  |
| SC vs. PC | 1.24 | 1.14-1.34 | <0.001 |
| MM vs. PC | 1.05 | 1.03-1.06 | <0.001 |
| MM vs. SC | 0.85 | 0.78-0.92 | <0.001 |
|  |  |  |  |
| *Time trend^c^* | 1.00 | 0.99-1.01 | 0.629 |
|  |  |  |  |
| *Season** |  |  |  |
| Summer | 1.00 |  |  |
| Winter | 1.01 | 0.91-1.13 | 0.795 |
| Spring | 1.16 | 1.04-1.29 | 0.009 |
| Autumn | 1.02 | 0.92-1.14 | 0.710 |
|  |  |  |  |
| ^a^ Level change refers to an abrupt level change of the Incidence rate between the periods; ^b^ Slope change refers to slope change of the incidence rate over time between the periods. ^c^Time trend refers to the change of Incidence rate associated with a time unit increase. Abbreviations: PED= Pediatric Emergency Department; PC= pre-COVID19 phase; SC= School closure phase; MM=Mitigation measures phase; HRR= Hospitalization Rate Ratio; 95%CI: 95% confidence interval. Note: MM vs. SC contrast was manually added for interpretative purposes without p-value adjustment for multiple comparison. | | | |

| Supplementary Table S11: Interrupted time series analysis results on PED attendance rates for Symptoms, Signs and Ill-defined Conditions, Injury and Poisoning, Diseases of the Respiratory System and Mental Disorders, excluding 0-1 years old subjects (sensitivity analysis). | | | | | | | | | | | | |
| --- | --- | --- | --- | --- | --- | --- | --- | --- | --- | --- | --- | --- |
|  | **Symptoms, Signs and Ill-defined Conditions** | | | **Injury and Poisoning** | | | **Diseases of the Respiratory System** | | | **Mental Disorders** | | |
|  | ***IRR*** | ***95%CI*** | ***p-Value*** | ***IRR*** | ***95%CI*** | ***p-Value*** | ***IRR*** | ***95%CI*** | ***p-Value*** | ***IRR*** | ***95%CI*** | ***p-Value*** |
| *Level change^a^* |  |  |  |  |  |  |  |  |  |  |  |  |
| SC vs. PC | 0.16 | 0.10-0.26 | <0.001 | 0.23 | 0.14-0.36 | <0.001 | 0.06 | 0.02-0.20 | <0.001 | 0.26 | 0.17-0.42 | <0.001 |
| MM vs. PC | 0.34 | 0.27-0.44 | <0.001 | 0.59 | 0.46-0.77 | <0.001 | 0.15 | 0.09-0.25 | <0.001 | 0.67 | 0.53-0.86 | 0.001 |
| MM vs. SC | 2.13 | 1.29-3.54 | 0.003 | 2.52 | 1.59-4.00 | <0.001 | 2.39 | 0.69-8.31 | 0.172 | 2.54 | 1.57-4.12 | <0.001 |
|  |  |  |  |  |  |  |  |  |  |  |  |  |
| *Slope change^b^* |  |  |  |  |  |  |  |  |  |  |  |  |
| SC vs. PC | 1.20 | 1.09-1.32 | <0.001 | 1.23 | 1.12-1.34 | <0.001 | 1.35 | 1.08-1.70 | 0.008 | 1.20 | 1.10-1.31 | <0.001 |
| MM vs. PC | 1.04 | 1.02-1.06 | <0.001 | 1.03 | 1.01-1.05 | 0.012 | 1.09 | 1.05-1.14 | <0.001 | 1.03 | 1.01-1.05 | 0.004 |
| MM vs. SC | 0.87 | 0.79-0.95 | 0.003 | 0.85 | 0.78-0.92 | <0.001 | 0.81 | 0.64-1.01 | 0.066 | 0.86 | 0.79-0.94 | 0.001 |
|  |  |  |  |  |  |  |  |  |  |  |  |  |
| *Time trend^c^* | 1.00 | 0.99-1.01 | 0.254 | 1.00 | 0.99-1.01 | 0.615 | 1.00 | 0.99-1.01 | 0.443 | 1.01 | 1.00-1.01 | 0.018 |
|  |  |  |  |  |  |  |  |  |  |  |  |  |
| *Season* |  |  |  |  |  |  |  |  |  |  |  |  |
| Summer | 1.00 |  |  | 1.00 |  |  | 1.00 |  |  | 1.00 |  |  |
| Winter | 1.33 | 1.18-1.50 | <0.001 | 0.72 | 0.63-0.82 | <0.001 | 1.76 | 1.39-2.23 | <0.001 | 0.89 | 0.78-1.02 | 0.086 |
| Spring | 1.26 | 1.11-1.43 | <0.001 | 1.10 | 0.98-1.25 | 0.114 | 1.39 | 1.08-1.78 | 0.010 | 0.98 | 0.86-1.13 | 0.821 |
| Autumn | 1.28 | 1.13-1.45 | <0.001 | 0.76 | 0.67-0.87 | <0.001 | 1.74 | 1.37-2.20 | <0.001 | 0.86 | 0.75-0.98 | 0.028 |
|  |  |  |  |  |  |  |  |  |  |  |  |  |
| ^a^ Level change refers to an abrupt level change of the Incidence rate between the phases; ^b^ Slope change refers to slope change of the incidence rate over time between the phases; ^c^ Time trend refers to the change of Incidence rate associated with a time unit increase. Abbreviations: PED= Pediatric Emergency Department; IRR: incidence rate ratio; PC= Pre-COVID19 phase; SC= School closure phase; MM=Mitigation Measures phase; 95%CI: 95% confidence interval. Pseudo R2=0.84 for Symptoms, Signs and Ill-defined Conditions, 0.77 for Injury & Poisoning model, 0.78 for Diseases of the Respiratory System model; 0.30 for Mental Disorders. Note: MM vs. SC contrasts were manually added for interpretative purposes without p-value adjustment for multiple comparison. | | | | | | | | | | | | |
