# Supplementary material for: Effects of COVID-19-targeted non-pharmaceutical interventions on pediatric emergency department use: a quasi-experimental study interrupted time-series analysis in North Italian hospitals, 2017 to 2022
Source: Front Public Health. 2024 Jul 31;12:1439078. doi: 10.3389/fpubh.2024.1439078 (PMC11322479; doi:10.3389/fpubh.2024.1439078)
Supplement: Supplementary file 2 [file Table_2.DOCX]

**SUPPLEMENTARY APPENDIX**

**LITERATURE REVIEW**

*RELEVANT STUDIES APPLYING INTERRUPTED-TIME SERIES (ITS) METHODOLOGY*

We reviewed the literature to identify existing studies investigating the effects of the pandemic on pediatric Emergency Department admissions, using Interrupted-Time Series (ITS) methodology.

An information specialist developed a search strategy for the PubMed database, in close collaboration with the whole research team. It was decided to omit terms concerning non-pharmacological interventions, since they are not always mentioned in the title or abstract of potentially relevant studies, which sometimes generically refer to consequences of the pandemic.

The strategy comprises four elements: COVID-19, ITS analysis, pediatric population, and emergency medicine.

PUBMED STRING ADOPTED:

("COVID-19"[Title/Abstract] OR "COVID-19"[MeSH Terms] OR "sars-cov-2"[Title/Abstract] OR "sars-cov-2"[MeSH Terms] OR "Severe Acute Respiratory Syndrome Coronavirus 2"[Title/Abstract]) AND ("child"[MeSH Terms] OR "child*"[Title/Abstract] OR "Pediatric Emergency Medicine"[MeSH Terms] OR "pediatric"[Title/Abstract] OR "paediatric"[Title/Abstract] OR "adolescent"[MeSH Terms] OR "adolescent*"[Title/Abstract] OR "young"[Title/Abstract] OR "teenager*"[Title/Abstract]) AND ("Emergency Medicine"[MeSH Terms] OR "emergency service, hospital"[MeSH Terms] OR "emergency services, psychiatric"[MeSH Terms] OR "emergency"[Title/Abstract]) AND ("Interrupted Time Series Analysis"[MeSH Terms] OR "interrupted-time-series"[Title/Abstract] OR "interrupted-time-series"[Title/Abstract] OR "Segmented linear regression models"[Title/Abstract])

Search run on March 27^th^ yielded 68 hits, of which 39 were eligible for our review, as presented below.

| **PMID** | **Title** | **Authors** | **Citation** | **Country** | **Age of included population in years** | **Conditions investigated (primary endpoint)** | **Setting** | **Single/multicenter** | **Institutions (general, specialized, e.g. psychiatric hospital)** | **Periods studied** | **Limit 1: focused on specific diagnoses** | **Limit 2: inappropriate age range** | **Limit 3: single-center** | **Limit 4: Follow-up <2 years** |
| --- | --- | --- | --- | --- | --- | --- | --- | --- | --- | --- | --- | --- | --- | --- |
| 37722746 | Emergency department visits and hospital admissions for suicidal ideation, self-poisoning and self-harm among adolescents in Canada during the COVID-19 pandemic | Poonai N, Freedman SB, Newton AS, Sawyer S, Gaucher N, Ali S, Wright B, Miller MR, Mater A, Fitzpatrick E, Jabbour M, Zemek R, Eltorki M, Doan Q | CMAJ. 2023 Sep 18 | Canada | 10 to 18 | suicidal ideation, self-poisoning and self-harm | ED and hospital | Multicenter | We obtained data from the Canadian Institute for Health Information on emergency department visits and hospital admissions from April 2015 to March 2022 among adolescents aged 10-18 years in Canada | We used interrupted time-series methods to compare changes in levels and trends of these outcomes between the prepandemic (Apr. 1, 2015-Mar. 1, 2020) and pandemic (Apr. 1, 2020-Mar. 31, 2022) periods | **Yes** | **Yes** | No | No |
| 37708644 | Counts and child protection reports of diagnosed child maltreatment before and after the COVID-19 pandemic onset | Rebbe R, Reddy J, Huang JS, Kuelbs CL, Putnam-Hornstein E. | Child Abuse Negl. 2023 Sep 12 | USA | <18 | Child maltreatment | ED and hospital | Single-center | Pediatric hospital | Between 2016 and November 2021 | **Yes** | No | **Yes** | **Yes** |
| 37484836 | Effect of the COVID-19 pandemic on emergency department attendances for pediatric injuries in Mozambique's central hospitals: an interrupted time series and a comparison within the restriction periods between 2019 and 2020 | Amado V, Moller J, Couto MT, Wallis L, Laflamme L. | Trauma Surg Acute Care Open. 2023 Jul 14 | [Mozambique](https://pubmed.ncbi.nlm.nih.gov/37484836/) | <=14 | Injuries | ED | Multicenter | Central hospitals | An interrupted time series was applied to weekly data of pediatric injuries from the ED records of four central hospitals in Mozambique in 2019 and 2020 | **Yes** | **Yes** | No | **Yes** |
| 37478513 | Child and adolescent psychiatric emergency admissions before, during and after the Covid-19 pandemic: An Interrupted time series analysis from Turkey | Kose S, Inal-Kaleli I, Boru E, Yavuz-Kan O, Hakverdi G, Ucuncu B, Calik HN, Savran-Yorulmaz S, Senturk-Pilan B, Ozbaran B, Isik H, Saz EU, Bildik T. | Asian J Psychiatr. 2023 Sep | Turkey | <=18 | Psychiatric | ED | Single-center | University hospital | January 2018 to December 2021 | **Yes** | No | **Yes** | **Yes** |
| 37437758 | Impacts of PM(2.5) before and after COVID-19 outbreak on emergency mental disorders: A population-based quasi-experimental and case-crossover study | Tao J, Yan J, Su H, Huang C, Tong S, Ho HC, Xia Q, Zhu C, Zheng H, Hossain MZ, Cheng J. | Environ Pollut. 2023 Oct 1 | China | Any age; no analysis for subgroup 0-18 years | Mental health | ED and hospital | Multicenter | Una città in Cina, non chiaro | 2016 to 2021.  A time-stratified case-crossover analysis was employed to evaluate the association of mental disorders with PM2.5 exposure before and after the COVID-19 outbreak | **Yes** | **Yes** | No | **Yes** |
| 37353150 | The Impact of COVID-19 on Infant Maltreatment Emergency Department and Inpatient Medical Encounters | Rebbe R, Reddy J, Kuelbs CL, Huang JS, Putnam-Hornstein E. | J Pediatr. 2023 Jun 22 | USA | 0 to 1 | Infant maltreatment | ED and hospital | Multicenter | single children's hospital system | January 1, 2016, through November 30, 2021, | **Yes** | **Yes** | No | **Yes** |
| 37297778 | Impact of the First Year of the COVID-19 Pandemic on Pediatric Emergency Department Attendance in a Tertiary Center in South Italy: An Interrupted Time-Series Analysis | Alongi A, D'Aiuto F, Montomoli C, Borrelli P. | Healthcare (Basel). 2023 Jun 2 | Italy | <=18 | Overall and cause-specific PED attendance | [ED](https://pubmed.ncbi.nlm.nih.gov/37297778/) | Single-center | Hospital | March-December 2020, which were compared with analogous intervals from 2016 to 2019; the pandemic period was divided into three segments: the "first lockdown" (FL, 9 March-3 May), the "post-lockdown" (PL, 4 May-6 November) and the "second lockdown" (SL, 7 November-31 December). | No | No | **Yes** | **Yes** |
| 37278275 | Quantifying the impact of the coronavirus 2019 pandemic on youth sports-related injuries in the USA | Oganezova K, Houlihan N, Maguire KJ, Lawrence JT, Ganley TJ, Williams BA. | J Pediatr Orthop B. 2023 Jun 5. doi: 10.1097/BPB.0000000000001096. Online ahead of print. | USA | <=19 | Sports-related injuries | ED | Multicenter | USA emergency departments | from 2016 to 2020 | **Yes** | No | No | **Yes** |
| 37131165 | Changes in emergency department utilisation in Germany before and during different phases of the COVID-19 pandemic, using data from a national surveillance system up to June 2021 | Schranz M, Boender TS, Greiner T, Kocher T, Wagner B, Greiner F, Bienzeisler J, Diercke M, Grabenhenrich L |  | Germany | Age in groups of 0–19 years, 20–39 years, 40–59 years, 60–79 years | ED utilization | ED | Multicenter | 20 emergency departments spread throughout Germany | For the pandemic period (16-03-2020 - 13-06-2021) four different phases of the COVID-19 pandemic were defined as interruption points, the pre-pandemic period (06-03-2017 - 09-03-2020) was used as the reference. | No | No | No | **Yes** |
| 36930732 | The Epidemiology of Pediatric Basketball Injuries Presenting to US Emergency Departments: 2011-2020 | Houlihan N, England P, Kiani SN, Ganley T. | Pediatr Emerg Care. 2023 Apr 1 | USA | <=19 | Basketball injuries | ED | Multicenter | Emergency departments | 2011-2020 | **Yes** | No | No | **Yes** |
| 36822810 | Use of mental health services in the aftermath of COVID-19 waves: a retrospective study conducted in a French Psychiatric and Neurosciences University Hospital | Perozziello A, Sousa D, Aubriot B, Dauriac-Le Masson V. | BMJ Open. 2023 Feb 23 | France | Age in groups of 0–14 years, 15–24 years, 25–44 years, 45–64 years >64 | Mental health | ED and hospital | Single-center | Psychiatric and Neurosciences University Hospital | 2019 to 2021 | **Yes** | **Yes** | **Yes** | **Yes** |
| 36787991 | The effect of the COVID-19 pandemic on pediatric asthma-related emergency department visits and hospital admissions in Montréal, Quebec: a retrospective cohort study | Chelabi K, Osmanlliu E, Gravel J, Drouin O, Tse SM. | CMAJ Open. 2023 Feb 14 | Canada | < 18 | Asthma | ED and hospital | Multicenter | tertiary pediatric emergency departments | Prepandemic (Jan. 1, 2017, to Mar. 31, 2020) and pandemic (Apr. 1, 2020, to June 30, 2021) periods. | **Yes** | No | No | **Yes** |
| 36643598 | Effect of the COVID-19 pandemic on the frequency of emergency department visits in Portugal: An interrupted time series analysis until July 2021 | Lopes S, Soares P, Santos Sousa J, Rocha JV, Boto P, Santana R. | J Am Coll Emerg Physicians Open. 2023 Jan 11 | Portugal | Any age. To model the pediatric subgroup we used the population of children and adolescents 0–17 years old | ED utilization | ED | Multicenter | Public hospitals | The prepandemic period corresponded to the months from January 2017 to February 2020 and the pandemic period to the months from March 2020 to July 2021. | No | **Yes** | No | **Yes** |
| 36541052 | Emergency Department Visits and Hospitalizations for Eating Disorders During the COVID-19 Pandemic | Milliren CE, Richmond TK, Hudgins JD. | Pediatrics. 2023 Jan 1 | USA | 10 to 19 | Eating disorders | ED and hospital | Multicenter | Pediatric hospitals | Pre- (January 2018-March 2020) and post-COVID-19 onset (April 2020-June 2022). | **Yes** | **Yes** | No | No |
| 36356971 | Respective roles of non-pharmaceutical interventions in bronchiolitis outbreaks: an interrupted time-series analysis based on a multinational surveillance system | Lenglart L, et all |  | 14 European countries | <1 | Bronchiolitis | ED | Multicenter | Paediatric emergency department of one of 27 centres | From January 2018 to March 2021 | **Yes** | **Yes** | No | **Yes** |
| 36289021 | The Effect of COVID-19 Stay-At-Home Orders on the Rate of Pediatric Foreign Body Ingestions | Neal JT, Monuteaux MC, Porter JJ, Hudgins JD. | J Emerg Med. 2022 Dec | USA | <=19 | Foreign body ingestions | ED | Multicenter | Data from the Pediatric Health Information System on more than 50 not-for-profit, tertiary care pediatric hospitals in the United States | We compared the visits for the 3 years prior to this date (pre–COVID-19 lockdown: March 1, 2017 to March 12, 2020) with the visits after this date (post–COVID-19 lockdown: March 13, 2020 to March 31, 2021). Although statewide lockdowns and school closures happened at different times, we thought that the date of | **Yes** | No | No | **Yes** |
| 36164850 | Suicide and Self-Harm in Youth Presenting to a US Rural Hospital During COVID-19 | Arakelyan M, Emond JA, Leyenaar JK. | Hosp Pediatr. 2022 Oct 1 | USA | 5 to 17 | Suicide or self-harm | ED and hospital | Single-center | Rural hospital | from January 1, 2017 to May 31, 2021. | **Yes** | **Yes** | **Yes** | **Yes** |
| 35939889 | The impact of stay-at-home orders on the rate of emergency department child maltreatment diagnoses | Negriff S, Huang BZ, Sharp AL, DiGangi M. | Child Abuse Negl. 2022 Oct | USA | <=18 | Child maltreatment | ED | Multicenter | large integrated healthcare delivery system in California | between January 1, 2019 and September 30, 2021. | **Yes** | No | No | **Yes** |
| 35836474 | Pediatric Mental Health Emergency Visits During the COVID-19 Pandemic | Hernández-Calle D, Andreo-Jover J, Curto-Ramos J, García Martínez D, Valor LV, Juárez G, Alcamí M, Ortiz A, Iglesias N, Bravo-Ortiz MF, Vega BR, Martínez-Alés G. | Scand J Child Adolesc Psychiatr Psychol. 2022 Jun 12 | Spain | < 18 | Mental health | ED | Single-center | Teaching hospital | October 2018 to April 2021, | **Yes** | No | **Yes** | **Yes** |
| 35818171 | Epidemiologic Changes in Pediatric Fractures Presenting to Emergency Departments During the COVID-19 Pandemic | Markiewitz ND, Garcia-Munoz J, Lilley BM, Oduwole S, Shah AS, Williams BA. | J Pediatr Orthop. 2022 Sep 1 | USA | 0 to 17 | Fractures | ED | Multicenter | National Electronic Injury Surveillance System (NEISS) database and the American Community Survey (ACS) | from 2016 to 2020 | **Yes** | No | No | **Yes** |
| 35698227 | A comparative analysis of pediatric mental health-related emergency department utilization in Montréal, Canada, before and during the COVID-19 pandemic | Beaudry G, Drouin O, Gravel J, Smyrnova A, Bender A, Orri M, Geoffroy MC, Chadi N. | Ann Gen Psychiatry. 2022 Jun 13 | Canada | 5 to 17 | Mental health | ED | Single-center | urban pediatric tertiary care hospital | between April 1, 2016 and November 30, 2021 | **Yes** | **Yes** | **Yes** | **Yes** |
| 35667622 | Mental Health Emergency Department Visits by Children Before and During the COVID-19 Pandemic | Shankar LG, Habich M, Rosenman M, Arzu J, Lales G, Hoffmann JA. | Acad Pediatr. 2022 Sep-Oct | USA | 5 to 17 | Mental health | ED | Multicenter | 10-hospital health system and a children's hospital in the Chicago area | from March 2018 to February 2021 | **Yes** | **Yes** | No | **Yes** |
| 35578918 | Pediatric Mental Health Presentations and Boarding: First Year of the COVID-19 Pandemic | Ibeziako P, Kaufman K, Scheer KN, Sideridis G. | Hosp Pediatr. 2022 Sep 1 | USA | ≤18 | Mental health and psychiatric boarding (Psychiatric boarding occurs when patients remain in emergency departments or are admitted to inpatient medical/surgical units to await placement at psychiatric treatment programs) | ED and hospital | Single-center | Pediatric hospital | the first 12 months of the pandemic (March 2019–February 2020) and the first 12 months of the pandemic (March 2020–February 2021). | **Yes** | Yes | **Yes** | **Yes** |
| 35551049 | Impact of COVID-19 pandemic on emergency department attendances for young people | Solanke F, Easton S, Selby A, James D, Roberts G. | Arch Dis Child. 2022 May 12:archdischild-2021-323389. doi: 10.1136/archdischild-2021-323389. Online ahead of print. |  | 0 to 24 | ED utilization | ED and hospital | Single-center | large emergency department (ED) and regional | (April 2016-February 2021). | No | **Yes** | **Yes** | **Yes** |
| 35502909 | Impact on emergency and elective hospital-based care in Scotland over the first 12 months of the pandemic: interrupted time-series analysis of national lockdowns | Shah SA, Mulholland RH, Wilkinson S, Katikireddi SV, Pan J, Shi T, Kerr S, Agrawal U, Rudan I, Simpson CR, Stock SJ, Macleod J, Murray JL, McCowan C, Ritchie L, Woolhouse M, Sheikh A. | J R Soc Med. 2022 Nov | Scotland | Any age. age group (<5, 5–14, 15–44, 45–64, 65–84 and 85+ years) | Accident and emergency attendances, and emergency and planned hospital admissions | ED and hospital | Multicenter | hospital NHS care provision at national level | (1) Before: weeks ending 28 March 2020 to 27 September 2020; and (2) After: weeks ending 4 October 2020 to 28 March 2021. | No | **Yes** | No | **Yes** |
| 35391746 | Shift in Clinical Profile of Hospitalized Pneumonia in Children in the Non-pharmaceutical Interventions Period During the COVID-19 Pandemic: A Prospective Multicenter Study | Rybak A, Ouldali N, Angoulvant F, Minodier P, Biscardi S, Madhi F, Hau I, Santos A, Bouvy E, Dubos F, Martinot A, Dommergues MA, Gras-Le Guen C, Launay E, Levieux K, Zenkhri F, Craiu I, Lorrot M, Gillet Y, Mezgueldi E, Faye A, Béchet S, Varon E, Cohen R, Levy C. | Front Pediatr. 2022 Mar 22 | France | 0 to 15 | Hospitalized community-acquired pneumonia | ED and hospital | Multicenter | pediatric emergency departments | Between June 2014 and December 2020, | **Yes** | **Yes** | No | **Yes** |
| 35266405 | Growth in emergency department self-harm or suicidal ideation presentations in young people: Comparing trends before and since the COVID-19 first wave in New South Wales, Australia | Sara G, Wu J, Uesi J, Jong N, Perkes I, Knight K, O'Leary F, Trudgett C, Bowden M. | Aust N Z J Psychiatry. 2023 Jan | Australia | 10 to 24 | self-harm or suicidal ideation presentations | ED | Multicenter | New South Wales emergency departments | Before COVID (2015 to February 2020) and since COVID (March 2020 to June 2021). | **Yes** | **Yes** | No | **Yes** |
| 34996305 | The impact of the COVID-19 pandemic on hospital-treated self-harm in Sydney (Australia) | Sperandei S, Page A, Bandara P, Reis A, Saheb R, Gaur P, Munasinghe S, Worne K, Fozzard C, Brakoulias V. | Aust N Z J Psychiatry. 2022 Nov | Australia | Any age. Age at presentation was categorised as: 0–14 years, 15–24 years, 25–44 years, 45–64 years and 65 years or older. | Hospital-treated self-harm | ED | Multicenter | Hospitals | January 2016 to June 2021 | **Yes** | **Yes** | No | **Yes** |
| 34981845 | Effects of public health measures during the SARS-CoV-2 pandemic on the winter respiratory syncytial virus epidemic: An interrupted time series analysis | Reyes Domínguez AI, Pavlovic Nesic S, Urquía Martí L, Pérez González MDC, Reyes Suárez D, García-Muñoz Rodrigo F. | Paediatr Perinat Epidemiol. 2022 May | Spain | 0 to 14 | respiratory syncytial virus acute bronchiolitis | ED and hospital | Single-center | University hospital | from January 2016 to February 2020 (pre-intervention period) and from March 2020 to June 2021 (post-intervention period) | **Yes** | **Yes** | **Yes** | **Yes** |
| 34832531 | Fall of Community-Acquired Pneumonia in Children following COVID-19 Non-Pharmaceutical Interventions: A Time Series Analysis | Rybak A, Yang DD, Schrimpf C, Guedj R, Levy C, Cohen R, Gajdos V, Tort J, Skurnik D, Ouldali N, Angoulvant F. | Pathogens. 2021 Oct 24 | France | <18 | Community-acquired pneumonia | ED | Multicenter | pediatric emergency departments | from 1 January 2017 to 31 December 2020 | **Yes** | No | No | **Yes** |
| 34781241 | Acute bronchiolitis and respiratory syncytial virus seasonal transmission during the COVID-19 pandemic in Spain: A national perspective from the pediatric Spanish Society (AEP) | Torres-Fernandez D, Casellas A, Mellado MJ, Calvo C, Bassat Q. | J Clin Virol. 2021 Dec | Spain | 0 to 2 | Acute bronchiolitis and respiratory syncytial virus | ED | Multicenter | Hospitals | between 1st January 2016 and 31st December 2020 | **Yes** | **Yes** | No | **Yes** |
| 34301264 | Trends of follow-up clinic visits and admissions three-months before and during COVID-19 pandemic at Tikur Anbessa specialized hospital, Addis Ababa, Ethiopia: an interrupted time series analysis | Abebe W, Worku A, Moges T, Tekle N, Amogne W, Haile T, Mekonen D, Habtamu A, Deressa W. | BMC Health Serv Res. 2021 Jul 23 | Ethiopia | age (under 5 years, 5–12 years, 13–44 years, 45–64 years, 65 years, and over) | Trends in hospital visits and admissions. Riportano anche accessi ED pediatrici | ED and hospital | Single-center | Hospital | between December 11, 2019, to June 7, 2020, | No | **Yes** | **Yes** | **Yes** |
| 34074990 | Effect of the COVID-19 Pandemic on Patient Volumes, Acuity, and Outcomes in Pediatric Emergency Departments: A Nationwide Study | Finkelstein Y, Maguire B, Zemek R, Osmanlliu E, Kam AJ, Dixon A, Desai N, Sawyer S, Emsley J, Lynch T, Mater A, Schuh S, Rumantir M, Freedman SB |  | Canada | <=18 | Pediatric ED utilization | ED | Multicenter | tertiary-care pediatric EDs | prepandemic (January 1, 2018-January 27, 2020), peripandemic (January 28, 2020-March 10, 2020), and early pandemic (March 11, 2020-April 30, 2020). | No | No | No | **Yes** |
| 34038928 | An Interrupted Time-Series Analysis of Pediatric Emergency Department Visits During the Coronavirus Disease 2019 Pandemic | Irvine MA, Portales-Casamar E, Goldman RD. | Pediatr Emerg Care. 2021 Jun 1 | Canada | <=18 | Pediatric ED utilization | ED | Single-center | Children's Hospital | between December 1, 2019, and August 31, 2020, and for 2 previous years. | No | No | **Yes** | **Yes** |
| 33782051 | Suicide attempt and intentional self-harm during the earlier phase of the COVID-19 pandemic in Washtenaw County, Michigan | Bergmans RS, Larson PS. | J Epidemiol Community Health. 2021 Oct | USA | 0 to >65; subgroup analysis: those aged 18–65 years versus those <18 years | Suicide attempt and intentional self-harm | ED and hospital | Single-center | regional tertiary academic medical centre | from October 2015 through October 2020 | **Yes** | No | **Yes** | **Yes** |
| 32838393 | The impact of public health interventions on critical illness in the pediatric emergency department during the SARS-CoV-2 pandemic | Dean P, Zhang Y, Frey M, Shah A, Edmunds K, Boyd S, Schwartz H, Frey T, Stalets E, Schaffzin J, Vukovic AA, Reeves S, Masur T, Kerrey B. | J Am Coll Emerg Physicians Open. 2020 Aug 10 | USA | <=18 | Total patient visits (ED and urgent care), shock trauma suite (STS) volume, and measures of critical illness | ED | Single-center | tertiary pediatric emergency department | from December 31 through May 14 of 6 consecutive years (2015-2020) | No | No | **Yes** | **Yes** |
| 32702427 | Trends in Pediatric Emergency Department Utilization after Institution of Coronavirus Disease-19 Mandatory Social Distancing | Chaiyachati BH, Agawu A, Zorc JJ, Balamuth F. | J Pediatr. 2020 Nov | USA | <=21 | ED utilization | ED | Single-center | tertiary urban children's hospital | comparing 2020 (the study time period) to the same date range during 3 prior years (2017 through 2019, | No | No | **Yes** | **Yes** |
| 38405774 | Increased pediatric RSV case counts following the emergence of SARS-CoV-2 are attributable to increased testing. | Petros BA, Milliren CE, Sabeti PC, Ozonoff A. | medRxiv [Preprint]. 2024 | USA | <18 | Respiratory syncytial virus (RSV) | ED, hospital, ICU | Multicenter | Pediatric hospitals | July 1, 2013 - June 30, 2023 | **Yes** | No | No | No |
| 38086185 | Unintentional pediatric poisonings before and during the COVID-19 pandemic: A population-based study. | Myran DT, Gaudreault A, McCarthy SDS, Pugliese M, Tanuseputro P, Finkelstein Y. | Am J Emerg Med. 2024 | Canada | 0 to 9 | ED visits and hospitalizations for poisonings | ED, hospital | Multicenter | Ontario population, health administrative data | pre-pandemic (January 2010-March 2020) and pandemic (April 2020-December 2021). | **Yes** | **Yes** | No | **Yes** |
