# Supplementary figures and images for: Effects of COVID-19-targeted non-pharmaceutical interventions on pediatric emergency department use: a quasi-experimental study interrupted time-series analysis in North Italian hospitals, 2017 to 2022
Source: Front Public Health. 2024 Jul 31;12:1439078. doi: 10.3389/fpubh.2024.1439078 (PMC11322479; doi:10.3389/fpubh.2024.1439078)

## Slide 1
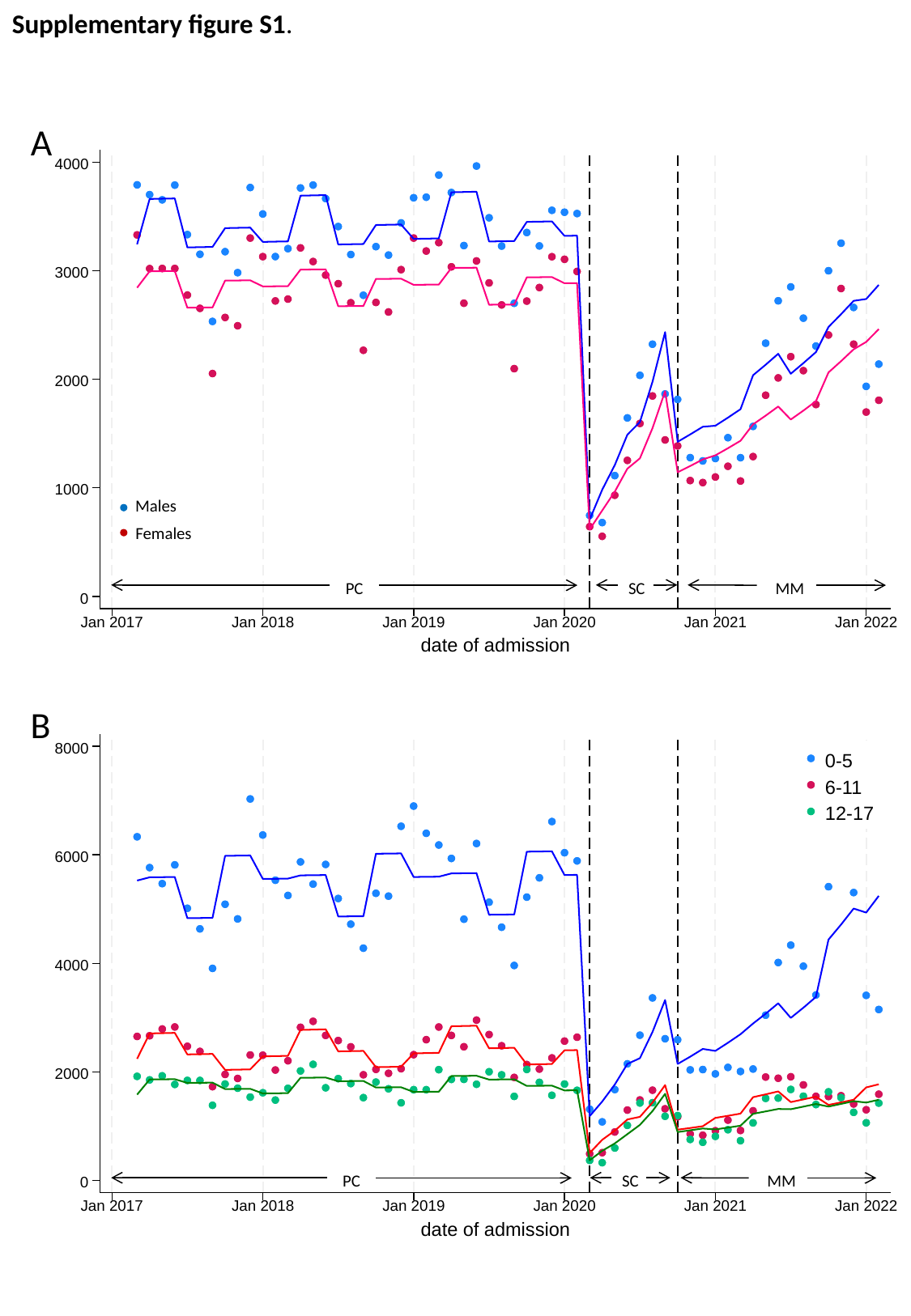

Supplementary figure S1.
A
Males
Females
MM
SC
PC
B
MM
SC
PC

Supplement: SUPPLEMENTARY FIGURE S1 — Monthly rate of PED attendance rate, with line trend from with line trend from Interrupted Time Series (ITS) regression analysis during the three time phases: the three years pre-COVID19 phase (PC), the subsequent one year “school closure” phase (SC, March to September 2020), and the one year “mitigation measures” phase (MM, October 2020 to February 2022), when schools were reopened but milder restrictions remained, by sex (A) and age category (B). PED, Pediatric Emergency Department; PC, pre-COVID19 phase; SC, School closure phase; MM, Mitigation measures phase. [file Presentation_1.PPTX]

## Slide 1
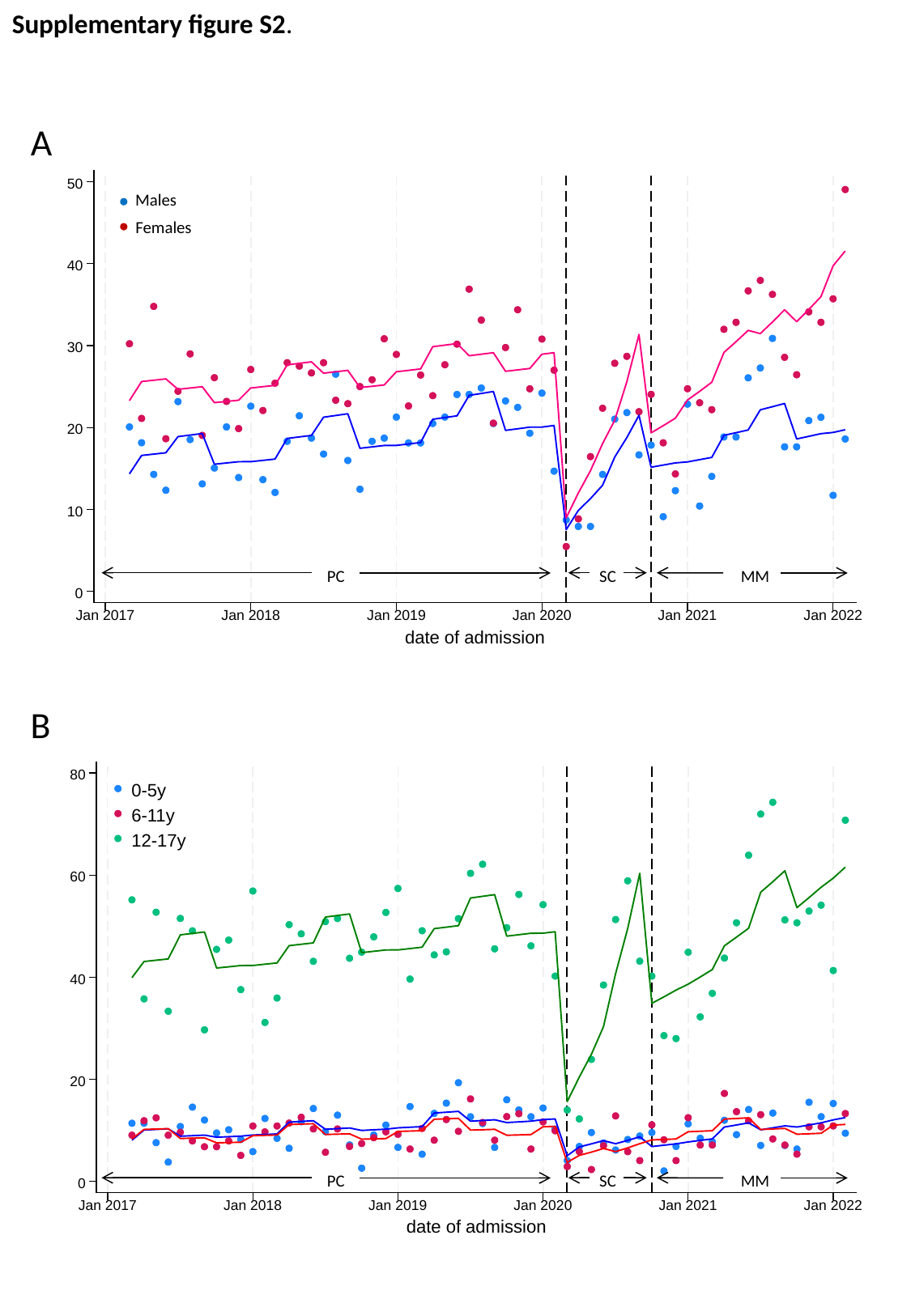

Supplementary figure S2.
A
Males
Females
MM
SC
PC
B
MM
SC
PC

Supplement: SUPPLEMENTARY FIGURE S2 — Monthly rate of PED attendance rate for Mental Disorders with line trend from Interrupted Time Series (ITS) regression analysis during the three time phases: the three years pre-COVID19 phase (PC), the subsequent one year “school closure” phase (SC, March to September 2020), and the one year “mitigation measures” phase (MM, October 2020 to February 2022), when schools were reopened but milder restrictions remained, by sex (A) and age category (B). PED, Pediatric Emergency Department; PC, pre-COVID19 phase; SC, School closure phase; MM, Mitigation measures phase. [file Presentation_2.PPTX]
